# Designing a rapid response program to support evidence-informed decision-making in the Americas region: using the best available evidence and case studies

**DOI:** 10.1186/s13012-016-0472-9

**Published:** 2016-08-18

**Authors:** Michelle M. Haby, Evelina Chapman, Rachel Clark, Jorge Barreto, Ludovic Reveiz, John N. Lavis

**Affiliations:** 1 Department of Chemical and Biological Sciences, Universidad de Sonora, Hermosillo, Sonora México; 2 Centre for Health Policy, Melbourne School of Population and Global Health, The University of Melbourne, Victoria, Australia; 3 Pan American Health Organization, Brasilia, DF Brazil; 4 Centre of Excellence in Intervention and Prevention Science, Melbourne, Australia; 5 Fundação Oswaldo Cruz, Diretoria de Brasília, Brazil; 6 Knowledge Management, Bioethics and Research, Pan American Health Organization, Washington, DC USA; 7 McMaster Health Forum, Centre for Health Economics and Policy Analysis, Department of Clinical Epidemiology and Biostatistics, and Department of Political Science, McMaster University, Hamilton, Canada; 8 Department of Global Health and Population Boston, Harvard T.H. Chan School of Public Health, Boston, MA USA

## Abstract

**Background:**

The objective of this work was to inform the design of a rapid response program to support evidence-informed decision-making in health policy and practice for the Americas region. Specifically, we focus on the following: (1) What are the best methodological approaches for rapid reviews of the research evidence? (2) What other strategies are needed to facilitate evidence-informed decision-making in health policy and practice? and (3) How best to operationalize a rapid response program?

**Methods:**

The evidence used to inform the design of a rapid response program included (i) two rapid reviews of methodological approaches for rapid reviews of the research evidence and strategies to facilitate evidence-informed decision-making, (ii) supplementary literature in relation to the “shortcuts” that could be considered to reduce the time needed to complete rapid reviews, (iii) four case studies, and (iv) supplementary literature to identify additional operational issues for the design of the program.

**Results:**

There is no agreed definition of rapid reviews in the literature and no agreed methodology for conducting them. Better reporting of rapid review methods is needed. The literature found in relation to shortcuts will be helpful in choosing shortcuts that maximize timeliness while minimizing the impact on quality. Evidence for other strategies that can be used concurrently to facilitate the uptake of research evidence, including evidence drawn from rapid reviews, is presented. Operational issues that need to be considered in designing a rapid response program include the implications of a “user-pays” model, the importance of recruiting staff with the right mix of skills and qualifications, and ensuring that the impact of the model on research use in decision-making is formally evaluated.

**Conclusions:**

When designing a new rapid response program, greater attention needs to be given to specifying the rapid review methods and reporting these in sufficient detail to allow a quality assessment. It will also be important to engage in other strategies to facilitate the uptake of the rapid reviews and to evaluate the chosen model in order to make refinements and add to the evidence base for evidence-informed decision-making.

**Electronic supplementary material:**

The online version of this article (doi:10.1186/s13012-016-0472-9) contains supplementary material, which is available to authorized users.

## Background

While research evidence is only one of many inputs into decision-making when it comes to health policy [1], it is important to try to maximize its usefulness and uptake. A range of programs and efforts already exist to promote the uptake of research evidence into policy and practice. These include efforts to conduct systematic reviews of the evidence (e.g., the Cochrane Collaboration) as well as efforts to package research evidence, including systematic reviews to inform policy and practice (e.g., Evidence-Informed Policy Network (EVIPNet), Health Technology Assessment agencies). However, while extremely useful, these programs are often not able to provide access to research quickly nor answer specific policy questions in a timely way.

There is a wide literature describing the barriers and facilitators to the consideration of research evidence in decision-making that can inform the design of a rapid response program to address this gap [2–6]. The most frequently reported barriers to evidence uptake are poor access to good quality relevant research and the lack of timely and relevant research output [5, 7]. The most frequently reported facilitators are collaboration between researchers and policymakers, improved relationships and skills [5], and research that accords with the beliefs, values, interests or practical goals, and strategies of decision-makers [8].

### What is a rapid response program and what types of programs already exist?

A rapid response program that provided rapid reviews of the results of high-quality research evidence, which was contextualized and targeted to the needs of decision-makers, with a fast turn-around time, and that included interaction between researchers and decision-makers could overcome some of the barriers and facilitate the uptake of research into policy and practice.

When describing and designing a “rapid response program,” it is important to consider (1) the product offered; (2) the strategies utilized to facilitate the uptake of the product into decision-making; and (3) how the program is operationalized to ensure that it runs smoothly. These three things together will determine if the rapid response program meets its intended purpose, i.e., to facilitate the use of high-quality research in health decision-making.

The number of rapid response programs offering such a service is increasing [9, 10]. The authors of a recent study surveyed 29 rapid response programs internationally [10]. Within, and across, these programs, there was a wide variation in the program objectives, types of questions answered, and processes and methods used [10]. The primary objectives for producing rapid reviews (the main product of rapid response programs) reported by respondents were to inform decision-making with regard to funding health care technologies, services, and policy and program development [10]. The rapid reviews answered many types of questions—clinical effectiveness (55.2 %), clinical efficacy (41.4 %), cost-effectiveness and/or cost savings (41.4 %), and safety (31.0 %)—and were used to support clinical practice guideline preparation (17.2 %) for either a health care technology or service. Some rapid response programs focused exclusively on questions centered on health system interventions, health services delivery, health care policy, coverage of a technology, operational efficiency, and quality improvement. Two organizations focused on specific health topics [10].

### What are the main products of rapid response programs?

The types of products produced by rapid response programs are widely varied [10, 11] but were categorized in a recent study based on the extent of synthesis [11]. Four product types were described: (1) inventories, which simply list what evidence is available; (2) rapid responses, which present the best available evidence but with no formal synthesis; (3) rapid reviews, which synthesize the quality of and findings from the evidence; and (4) automated approaches, which generate meta-analyses in response to user-defined queries [11].

We focused our research on products that include a synthesis of the evidence because this is the most widely researched product and most comparable to a standard systematic review and because we consider it to be the most useful for increasing the use of research in health decision-making. This type of rapid product has many names, including rapid review, evidence summary, brief review, rapid systematic review, and rapid health technology assessment. In this paper, we have used the term “rapid review” as it is the most widely used term in the published literature [12, 13].

We understand a rapid review to be “a type of systematic review in which components of the systematic review process are simplified, omitted or made more efficient in order to produce information in a shorter period of time, preferably with minimal impact on quality. Further, rapid reviews typically involve a close relationship with the end-user and are conducted with the needs of the decision-maker in mind” (Haby MM, Chapman E, Clark R, Barreto J, Reveiz L, Lavis JN.: What are the best methodologies for rapid reviews of the research evidence for evidence-informed decision making in health policy and practice: a rapid review, submitted).

### Objective of this research

The objective of this work was to inform the design of a rapid response program to support evidence-informed decision-making in the Americas region. Because the design of the program could vary depending on the level of decision-making (e.g., country, state, municipality), the setting, the resources available (financial and workforce), and the needs of decision-makers, we offer guidance on factors that would need to be taken into account in setting up a rapid response program in the Americas region. In practice, the work is also likely to be relevant to other regions of the world. Specifically, we set out to answer the following three questions:

1. What are the best methodological approaches for rapid reviews of the research evidence (the product)?
2. What other strategies are needed to facilitate evidence-informed decision-making in health policy and practice?
3. How best to operationalize the program?

## Methods

The evidence used to inform the design of a rapid response program included the following: (i) two rapid reviews of the best available research evidence (for questions 1 and 2), (ii) supplementary literature in relation to the “shortcuts” that could be considered to reduce the time needed to complete rapid reviews (question 1), and (iii) four case studies and supplementary literature to identify operational issues for the design of the program (question 3).

### Rapid reviews

We conducted two rapid reviews of the research evidence to answer the questions:

1. What are the best methodologies to enable a rapid review of research evidence for evidence-informed decision-making in health policy and practice?
2. What are the best strategies to facilitate evidence-informed decision-making in health policy and practice?

Both reviews utilized systematic review methods and were conducted according to a pre-defined protocol, including clear inclusion criteria [14]. Comprehensive search strategies were used, including published and gray literature, written in English, French, Portuguese, or Spanish, from 2004 onwards. Eleven databases and two websites were searched. Two review authors independently applied eligibility criteria. Data extraction was done by one reviewer and checked by a second reviewer. The methodological quality of included studies was assessed independently by two reviewers. A narrative summary of the results is presented. Full details of the methods, including the inclusion criteria, can be found in an associated report [15] and paper (Haby MM, Chapman E, Clark R, Barreto J, Reveiz L, Lavis JN.: What are the best methodologies for rapid reviews of the research evidence for evidence-informed decision making in health policy and practice: a rapid review, submitted) for question 1 and in Additional files 1 and 2 for question 2. We have labelled these reviews as rapid reviews because they were conducted in a limited timeframe and with the needs of the decision-makers in mind.

### Identifying supplementary evidence for potential shortcuts

In relation to areas where “shortcuts” could be considered to reduce the time needed to complete the rapid reviews, we used the systematic reviews included in the previously mentioned review of methodologies of rapid reviews and the A MeaSurement Tool to Assess systematic Reviews (AMSTAR) questions [16] to make a list of all possible areas (shown in column 2, Table 1). To determine the potential impact of the shortcut on the validity of the results, we used the primary studies included in the reviews by Ganann and colleagues [17] and Cameron and colleagues [18] and complemented them with studies cited in the Cochrane Handbook [19] or found during the search process for the rapid review. It is important to note that this was not a systematic search for evidence, though the majority of the references come from the systematic review by Gannan and colleagues.

**Table 1:** Areas where “shortcuts” could be considered to reduce time to completion of rapid reviews

Systematic review step	Possible “shortcuts”	Potential impact on the validity of the results	Relevant AMSTAR question and potential impact of shortcut on AMSTAR score
Preparation of a protocol	• Omit protocol	Unknown	Q1. Loss of one point if a protocol is not prepared and/or not mentioned in report
Question formulation	• Limit the number of questions and sub-questions • Limit the scope of the question/s	None expected	
Selecting relevant studies	• One reviewer screens titles and abstracts • One reviewer screens full text	Unknown, though one reviewer could miss up to 9 % of eligible randomized controlled trials [42]	Q2. Loss of one point if only one reviewer does screening and/or only one reviewer does data extraction
Data extraction	• One reviewer extracts data	Can increase the number of errors but the impact on results is not known [43–45]	
	• One reviewer extracts data with checking by a second reviewer	Unknown	
	• Data extraction limited to key characteristics, results, conflicts of interest	Unknown	
Literature search	• Limit number of databases searched • Limit or omit hand searching of references lists and relevant journals • Eliminate consultation with experts to find additional studies	Limiting the number of databases searched can increase efficiency without compromising validity [46–51], especially if combined with some hand searching and contact with experts [52–57]	Q3. Loss of one point if less than two databases searched and/or no supplementary strategies
Inclusion criteria			
Gray literature	• Limit or omit gray literature	Could introduce publication bias but the evidence is mixed [16, 58–60]	Q4. Loss of one point if gray literature omitted
Language	• English only	Effect can vary depending on the question [50, 61–68]	
Dates	• Narrow time frame, e.g., last 5 or 10 years	None expected	
Study types	• Restrict study types to systematic reviews (and economic evaluations) • Restrict study types to randomized controlled trials or controlled clinical trials (and economic evaluations)	None expected [69–72]	
Quality assessment	• Limit or omit quality assessment	Not recommended. Several authors suggest that, where resources are limited, priority should be given to quality assessment rather than extensive searching [51, 73]	Q7 and Q8. Loss of two points if not assessed, documented and used in formulation of conclusions
	• Omit “a priori” specification • Done by one reviewer	Unknown	
Data synthesis	• Narrative synthesis only (no meta-analysis)	Unknown – meta-analysis can increase power and precision but also has potential to mislead if not applied appropriately and done correctly [19]	Q9. None if explained that meta-analysis not possible due to heterogeneity. If not, loss of one point
Assessment of publication bias	• Omit	Unknown	Q10. Loss of one point if omitted
Assessment of conflict of interest	• Omitted for individual studies and/or for systematic review	Unknown	Q11. Loss of one point if omitted
Report	• Information included limited	Unknown but can impact on AMSTAR score if insufficient detail of methods provided to enable a quality assessment. Sufficient detail of methods will help the reviewer to assess the validity of the results [17, 18, 23]	Q1–11. Potential large loss of points if key AMSTAR questions not covered
External peer review	• Omit or limit	Unknown	

### Case studies of existing rapid response programs

We developed cases studies of existing rapid response programs to highlight operational issues that were not addressed in the systematic reviews included in the review of methodologies for rapid reviews [9, 17, 18, 20–23]. Case studies are useful for identifying program processes, barriers and facilitators and can alert the practitioner to the existence of otherwise unexplored or unusual phenomenon [24]. The sampling frame for the selection of case studies was programs known to the authors and/or identified during the search process for the two rapid reviews. To select the four programs for case studies, we used purposeful sampling to identify four unique cases [25]. We aimed for maximum variation in terms of their reach (state level, national, or global) and stage of development (just starting or long history) and to ensure representation of both developed and developing countries. These case studies are not meant to be representative of all rapid response programs. The four programs chosen and the main reason/s for their selection are shown in Table 2.

**Table 2:** Rapid response programs selected for case studies

Rapid response program	Reason for selection	References
Cochrane response by Cochrane Innovations, Cochrane Collaboration^a^	Has a potential global reach and is supported by the expertise and experience of the Cochrane Collaboration, though is still in development	
McMaster Health Forum Rapid Response Program, McMaster University, Canada	Comes from a developed country with a strong history in knowledge translation, potentially national reach	[27, 28, 74]
Regional East African Community Health (REACH) Policy Initiative, Uganda	Comes from a low-income economy and has a published evaluation	[35]
Sax Institute Evidence Check Program, NSW, Australia	Has a long history (since 2007) and is a state-level program	[75, 76]

^a^Cochrane Innovations is a trading company wholly owned by The Cochrane Collaboration

Case studies were developed based on a structured interview with a key informant from the program, conducted using Skype. This information was supplemented with information on the program website and from the published literature. The questions used in the structured interview are included in Additional file 3. These were sent to the key informant prior to the interview. Key informants were also offered the chance to review the draft case study before publication. Our first request for participation was made to the most senior and knowledgeable representative of the organization offering the program. In two cases, we were referred to an alternative knowledgeable contact (McMaster Health Forum and Cochrane Response).

For one case study (Regional East African Community Health (REACH) Policy Initiative), the key informant agreed to participate in the interview but then did not make any further contact, despite various attempts on our part, including a request to review the draft case study. As an alternative, the informant could not be identified; we decided to develop the case study based on the information obtained from the published literature [26] and websites only. All three interviews were conducted in the first two weeks of June 2015. One key informant (from the Sax Institute) provided written answers to most of the questions prior to the interview, and this information was supplemented in the interview.

The answers to the interview questions were summarized in a one-page format (see Additional file 3). The general structure used was a two- to three-paragraph description of the program, including its scope, who it services, the length of time it has been operating, governance and operational arrangements, staffing, products, and process. This was followed by sections on documents available to guide the reviews, strengths of the program, challenges, and future work. The source of information is noted at the end. Where the program offered more than one product type, we focused on the product closest to a rapid review. Given the variation in set-up and conduct of the four programs, it was not possible to be too prescriptive about how the relevant elements were reported. The draft case study was checked by the key informant (with the exception of the REACH Policy Initiative as no response was received from them) and appropriate revisions made. For Cochrane Innovations, the CEO was also contacted, at the suggestion of the key informant, to comment on the case study but no comments were received.

### Supplementary literature to inform the design and operationalization of the program

In the process of conducting the rapid reviews, we identified a recent study by Hartling and colleagues that provides information gathered from interviews with key informants of 20 different rapid response programs [11]. As part of this study, they describe contextual factors that influenced rapid review methods. Other insights into how a rapid response program could be operationalized are presented by Wilson and colleagues based on findings from an issue brief and stakeholder dialogue conducted with health system decision-makers to inform the development of a rapid response program for Canada [27, 28].

## Results

### Question 1—methodologies for rapid reviews

#### Key findings from the rapid review

While five systematic reviews of methods for rapid reviews were found, none of these were of sufficient quality to allow firm conclusions to be made. Thus, the findings need to be treated with caution. There is no agreed definition of rapid reviews in the literature and no agreed methodology for conducting rapid reviews [9, 17, 18, 20–23]. However, the systematic reviews included in this review are consistent in stating that a rapid review is generally conducted in a shorter timeframe and may have a reduced scope. A wide range of “shortcuts” are used to conduct rapid reviews more quickly than a full systematic review. While authors of the included systematic reviews tend to agree that changes to scope or timeframe can introduce biases (e.g., selection bias, publication bias, and language of publication bias), they found little empirical evidence to support or refute that claim [9, 17, 18, 20, 22]. Further, there are few comparisons available in the literature of full and rapid reviews to be able to determine the impact of these “shortcuts.” There is some evidence from a good quality randomized controlled trial with low risk of bias that rapid reviews may improve clarity and accessibility of research evidence for decision-makers when compared to a systematic review alone [29].

The included systematic reviews included a variety of rapid products, including rapid systematic reviews, rapid health technology assessments, and rapid overviews of systematic reviews. However, no examples of rapid evidence briefs for policy were included in the reviews.

The authors of the published systematic reviews of rapid review methods suggest that, rather than focusing on developing a formalized methodology, which may not be appropriate, researchers and users should focus on increasing the transparency of the methods used for each review [17, 18, 23]. The authors of the most recent systematic review also suggest that: “the similarity of rapid products lies in their close relationship with the end user to meet decision-making needs in a limited timeframe” ([9], p. vii).

#### Key findings from supplementary literature in relation to ‘shortcuts’

The areas where “shortcuts” could be considered to reduce the time needed to complete the reviews are shown in Table 1. As can be seen from column 3 of the table, the supporting literature for the potential impact of shortcuts in the review process is quite limited and not always conclusive. It is important to note that not all shortcuts necessarily lead to a reduction in the AMSTAR score (e.g., limiting the scope of the review question) and some shortcuts can have significant implications for the AMSTAR score but not necessarily save a significant amount of time (e.g., omitting key methodological details from the report).

### Question 2—strategies to facilitate evidence-informed decision-making

#### Key findings from the rapid review

Forty systematic reviews of strategy effectiveness met the inclusion criteria for this question, of which data were extracted from 27 of the systematic reviews (see Additional files 1 and 2). Using the domains of the linking research to action framework [7, 30], the majority of the interventions that were evaluated in the included systematic reviews focused on the practice rather than the policy environments and, within the former, on “push,” “facilitating pull,” and “pull” activities. Examples of interventions with significant impact include dissemination of printed educational materials, including systematic reviews; clinical librarian services; education in evidence-based practice; local opinion leaders; and tailored and targeted messaging (Additional file 1). For linkage and exchange, knowledge brokers and interaction between users and producers of research are the only interventions evaluated but the included studies do not provide any evidence of effectiveness for these interventions.

In regard to rapid reviews, no primary studies of the effectiveness of strategies to facilitate their uptake were found by the systematic reviews’ authors. Further, there are no good quality evaluations of the impact on research use of packaging of systematic reviews as overviews of systematic reviews or as evidence briefs based on systematic reviews [31, 32]. However, there is evidence that dissemination of printed educational materials [33] and summaries of systematic reviews [32, 34] are effective at improving awareness and/or clinical practice (Additional file 1).

### Question 3—how best to operationalize the program

#### Key findings from the case studies

The case studies are presented in Additional file 3. The models presented in the case studies vary in terms of when they started, the reach of the service, how the service is funded, whether reviews undergo external review, and whether reviews are made publicly available (Table 3). Most of the models include a lag period before publication of the review to allow the commissioning agency time to prepare for any resulting publicity or to allow for journal publications to be submitted.

**Table 3:** Features of the four rapid response models developed as case studies

Model	Started	Reach	How funded	Time to complete a RR	External review of RR	RRs made publicly available	Lag period before publication
Cochrane response	2013	Potentially global	*Service* Not clear *Reviews* “User-pays”	≈8 weeks (first review took 12 weeks)	Yes	All	Yes
McMaster Health Forum Rapid Response Program	2012	Potentially national (Canada)	*Service* Ontario government *Reviews* Free in Ontario, “User-pays” rest of Canada	Max 6 weeks	Yes	All	Yes
REACH Policy Initiative	2010	National (Uganda)	*Service and reviews* Donor funds	Max 4 weeks	Yes	Not reported	Not reported
Sax Institute Evidence Check Program	2006	State, potentially national (Australia)	*Service* State government plus other funds *Reviews* “User-pays”	≈12–16 weeks	No	Most (some kept confidential if requested by funder)	Yes

*RR* rapid review

While all programs have some documentation to guide the process and methods, the actual methods used can vary between reviews conducted within the same program—depending on the question and needs of the requestor. The reporting templates used in the models vary in their prescriptiveness but none include minimum methodological standards or reporting requirements to enable an accurate quality assessment. For all models, the process involved in conducting the review requires regular interaction and communication between researchers and decision-makers. None of the models have needed to use priority setting to determine which reviews to undertake—all reviews that are within the scope of the service have been accepted.

Each of the models is unique in some way. The McMaster model is the only one that reported using other knowledge translation strategies associated with the conduct of the review, i.e. wide dissemination and promotion of the completed review, including on social media, and the offer of a presentation of the review’s findings to the requestor. The Sax Institute model is the only one that brokers the reviews out to research groups selected from their database of researchers or identified through other means. This model has the advantages of having researchers with specific expertise in the topic conduct the review and of building skills in writing for policy makers in a wider range of researchers. A potential disadvantage of this approach is the extra work required by the Sax Institute staff to ensure the review deadlines are met and to support new researchers (which is currently done through a one on one informal mentoring process). The Cochrane Innovations model is unique in having the support of a large group of highly skilled reviewers, though only one review has been conducted to date. The REACH-PI model is the first known example in a low-income country and is the only one with some published evaluation results [35]. Operational issues highlighted by the key informants or in the published evaluation [26] that need to be considered by developers of new programs are reported in Table 4.

**Table 4:** Operational issues highlighted by the case studies

Issue	Description of issue
Contracts and intellectual property	If it is a “user-pays” model the use of a contract can slow the process down. However, the impact can be minimized by operating in good faith and starting the review before the contract is signed (e.g. Cochrane Innovations, Sax Institute). Where a contract is used, the intellectual property is usually owned by the funder but there seems to be general acceptance of joint publication of completed reviews. However, this may not always be the case, for example, for the Sax Institute model not all reviews are made publicly available if confidentiality is requested by the funder.
External review of the rapid review	External review or “merit review” has the potential to slow the process down if reviewers don’t respond quickly but the different services all seem to have found ways to manage this well, e.g., approaching another reviewer if the first one can’t commit to a quick response.
Staffing	Recruiting staff with the right mix of skills and qualifications was noted as an issue for the REACH Policy Initiative model. The other three models used mentoring or internal training to address this issue, with the Sax Institute key informant noting that the Institute also had plans to develop a formal training program for researchers.
Evaluation	None of the models have formally evaluated the impact of the service on the uptake of research evidence for policy and/or practice—though there are plans to do this for the McMaster service [27].
Issues particular to developing countries	Having a fast and reliable internet connection was noted as an issue for the REACH Policy Initiative model. Access to databases and full text papers was noted as an issue for the REACH Policy Initiative model.

#### Key findings from supplementary literature

In relation to how rapid response programs are operationalized to ensure that they run smoothly and meet their intended purpose, the available literature is limited. The study by Hartling and colleagues describes contextual factors that influenced rapid review methods [11]. These were identified by thematic analysis of the interviews. Two of these contextual factors related to how the program was operationalized and are:

- The continuous close relationship with a specific end user in an iterative fashion throughout the work to ensure that the product will meet the end user’s need
- A high reliance on maintaining highly trained staff to conduct the reviews in a short time frame and that understand the type of product that might meet the needs of the decision-maker [11]

Other insights into how a rapid response program could be operationalized are presented by Wilson and colleagues based on findings from their issue brief and stakeholder dialogue [27, 28]. Wilson and colleagues were unable to find any systematic reviews that addressed how a rapid review program could be organized. Therefore, they based their insights on examples of existing rapid review programs [28]. In their issues brief they presented four organizational features and possible approaches to operationalizing each feature in Canada. The features included governance, management and staffing, program resources, and collaboration [27].

The following suggestions and issues raised by Wilson and colleagues for a Canadian program warrant consideration in setting up a rapid response program in other jurisdictions:

- Include high-level representation from all relevant stakeholders, including policymakers and researchers, on a steering committee to govern the service
- Implement minimum training standards and provide ongoing mentorship for staff contributing to the program
- Funding needs to be long-term and cover both program delivery and ongoing evaluation of the program
- Operationalization of the priority setting (if needed) and administrative process needs to be done at the institutional level [27, 28]

Two key challenges noted by the stakeholder dialogue participants when discussing the issues brief included securing stable, long-term funding and finding a way to effectively and equitably manage the expected demand [27].

### Insights for designing a rapid response program for the Americas region

#### The product — rapid reviews

The systematic reviews published on this topic advise against developing a formalized methodology but advocate for greater transparency regarding the methods used for each review [17, 18, 23]. The four case studies also showed that current models do not use a formalized, one-size-fits-all methodology. It is likely that this variation in methodology is due to the differing user requirements. Further, the rapid reviews produced by the four programs (and available on their website) do not consistently report clearly and in sufficient detail the methods used within their reviews to enable an accurate quality assessment.

None of the included systematic reviews or case studies explicitly included evidence briefs for policy as a rapid product. However, rapid reviews produced as part of a rapid response program could be used to inform evidence briefs for policy. Also, there is a potential for evidence briefs for policy to be conducted quickly as part of a rapid response program. However, the evidence base supporting this product is limited.

The ideal methods for evidence syntheses continue to be high quality systematic review methods. Thus, it is important that the authors of the rapid reviews are transparent in reporting the methods they used so that the “shortcuts” taken can be clearly seen and the quality of the resulting product evaluated. The current, or modified, PRISMA Guidelines are a good starting point for this [36]. This will also enable researchers to evaluate the impact of methodological choices on the results of the reviews, whether rapid or full systematic reviews or overviews of systematic reviews.

Given that rapid reviews are often (though not always) written up in a concise fashion to meet the needs of busy decision-makers, a checklist could be developed that lists the key aspects of systematic review methodology and requires a simple tick or cross to indicate whether or not it was done (e.g., two reviewers screened titles and abstracts). For some aspects, a short answer would be appropriate, e.g., which databases were searched. Table 1 will be a very useful reference for this. If preferred, the methods section could be included at the end of the review, as an appendix to the review or placed online. Further, it is appropriate to evaluate rapid reviews using the same criteria as systematic reviews, e.g., using the AMSTAR criteria [16], which will also allow better comparisons with systematic reviews on issues of validity of the results. We suggest that the AMSTAR criteria could be used to assess the quality of reviews of systematic reviews (also known as overviews) as well as reviews of primary studies.

It is possible that the greater the number of “shortcuts” that are taken to reduce the time needed to complete the review the greater the risk that biases will be introduced. Therefore, an appropriate balance needs to be found between quality and timeliness when deciding what methods to use, whether a rapid (vs full systematic) review is needed and how quickly it really is needed.

Consideration also needs to be given to which “shortcuts” are taken to ensure maximum quality. Some shortcuts may be seen as more important by users in terms of their perceived impact on risk of bias of the results. We suggest that omission of the quality assessment of included studies could be one such shortcut, as implied by participants’ ranking of six rapid review approaches in the study by Tricco and colleagues [13, Table 5], where the top ranked approach included a quality assessment. Its omission will also lead to a loss of two points on the AMSTAR score (Table 1). In contrast, limiting the number of databases searched to to to three and only including the last 10 years of literature may have limited impact on perceived risk of bias for many topics and no impact on the AMSTAR score.

As well as the shortcuts identified in Table 1, other approaches exist that can be used to make a rapid review faster than a systematic review. These should not have an impact on the validity of the results and include [20]:

- Making the process more efficient, e.g., using specialized software for the reviews such as DistillerSR®
- Using a larger, highly skilled staff, who are part of a reserve capacity
- Updating an existing high quality review

#### Strategies to facilitate evidence-informed decision-making

Given that there is no support for the effectiveness of rapid reviews alone in promoting the uptake of research evidence into policy and practice (Additional file 1), it will be extremely important to also engage concurrently in other knowledge translation strategies as part of the model. Examples include the use of tailored and targeted messaging in the rapid reviews, dissemination of the rapid reviews, and training of policy makers in the appraisal and use of research (Additional file 1). It will also be important to evaluate the impact of these strategies to improve the evidence base for future decision-making regarding the design of rapid response programs.

It is important to remember that rapid reviews are typically conducted at the request of a decision-maker and the decision-maker has an important involvement in setting the question, the parameters of the review and the timeline. They may also provide feedback along the way. And unlike traditional systematic reviews, they are usually written with the context of the decision-maker in mind, e.g., the policy or practice question that needs an answer [9]. We suggest that these features are likely to increase the chance that the rapid reviews are utilized in decision-making but this needs to be tested.

#### Operationalization of the rapid response program

In regard to the organizational arrangements for the rapid response program, consideration will need to be given to the issues raised in other related literature presented in this paper [11, 27, 28] (see the “Results, Question 3—how best to operationalize the program” section) or in the case studies (Table 4).

## Discussion

The research presented in this paper will help to inform the design of a rapid response program for health policy and practice that can be applied to the Americas region. The rapid review of methodologies for rapid reviews and additional evidence found in relation to shortcuts that could be considered to reduce the time needed to complete the reviews (Table 1) are relevant to the design of the main product of the rapid response program—the rapid review. The rapid review of strategies to facilitate evidence-informed decision-making found a number of strategies that had a significant impact and which could be incorporated into the program. Finally, the case studies of four current models of rapid response identified additional operational issues that need to be considered in designing new programs (Table 4), as did the supplementary literature [11, 27, 28].

### Implications for future research

Priority for future research should be given to research into the effect of shortcuts on the conclusions of reviews in general (rapid and systematic)—thus filling the gaps in Table 1, column 3. Further, we suggest that research that compares the conclusions of systematic reviews and rapid reviews for the same topic, controlling for review quality, should also be prioritized.

We agree with Polisena and colleagues that the impact of rapid reviews used to inform health decision-making should be tested, as should the feasibility and desire for an extension to the PRISMA guidelines to incorporate rapid review methodologies, including different types of reports needed [10]. We suggest that the content of revised PRISMA guidelines should be heavily based on the current guidelines but allow for different ways to present the information, including in appendices or using checklists. They should also focus on aspects that are not routinely part of systematic reviews, such as the extent of involvement of the review funders in the conduct of the review as this could impact on quality (e.g., potential conflict of interest) but could also facilitate the use of the rapid review in decision-making.

Current reviews of rapid review programs suggest that one of the defining features of rapid reviews (and other rapid products) is the close relationship with the end user [9], with personal contact between researchers and decision-makers being a known facilitator of the uptake of evidence [2, 5, 37]. The process can also potentially build skills [5] and trust [37] between researchers and decision-makers. Research is required to identify which elements of this relationship increase the use of the review in decision-making (if they do). This research should also be extended to test the impact of these strategies in increasing the uptake of research in general, including systematic reviews, in decision-making.

### Strengths and limitations

A key strength of this research was the use of high quality systematic review methods to guide the development of a rapid response program as well as case studies and further literature searching to help fill knowledge gaps. The case studies were able to verify the large variation in existing programs [9–11, 13, 18, 23] and identify operational issues that had not been previously identified. A limitation of our research is the small number of case studies included, but this was supplemented with the findings of more comprehensive surveys of current rapid response programs [9–11, 13] (see the “Background” section) and other relevant work in this area [27, 28].

Another possible limitation of our research and insights offered for a rapid response program is the reliance on AMSTAR for assessing and comparing the quality of rapid reviews and systematic reviews. AMSTAR was created to assess the quality of systematic reviews of randomized controlled trials, and thus, it could be argued that it is not appropriate for other types of reviews, e.g., reviews of reviews, reviews of non-randomized studies, and realist reviews. At the present time, however, the AMSTAR tool is the best tool available to our knowledge to assess and compare the quality of review methods for reviews of effectiveness and considers the major potential sources for bias in reviews of the literature [16, 38]. As other tools are developed and more widely used, such as RAMESES for realist reviews [39], variations of AMSTAR for non-randomized studies [40], and the new ROBIS tool to assess the risk of bias in systematic reviews [41], they can also be used for rapid response programs to assess the quality of their products.

## Conclusions

There is no one clear method or process for rapid response suggested by the research evidence found in the rapid reviews, related literature, or case studies—and a variety of approaches may be needed. The ideal methods for evidence synthesis continue to be high quality systematic review methods. However, the literature, and our experience, tells us that policy and practice decisions need to be made and cannot always wait until the best evidence is available. In these cases, there is a very real risk that no research evidence or poor quality research evidence will be used. Thus, it is better that researchers try to accommodate the needs of busy decision-makers by producing rapid reviews that optimally balance timeliness and quality and, where possible, to evaluate later how the results differ from full reviews. In this paper, we have highlighted factors that need to be taken into account in setting up a rapid response program. We also advocate for greater attention to be given to the reporting of methods used in rapid reviews to allow a quality assessment. Finally, it will be important to evaluate the rapid response programs chosen model in order to make refinements and to add to the evidence base for evidence-informed decision-making.

## Additional files

Additional file 1: What are the best strategies to facilitate evidence-informed decision-making: a rapid overview of systematic reviews. (DOCX 323 kb)

Additional file 2: Appendices to the rapid overview of strategies to facilitate evidence-informed decision-making. (DOCX 187 kb)

Additional file 3: Case studies. (DOCX 40 kb)
