# Supplementary material for: Designing a rapid response program to support evidence-informed decision-making in the Americas region: using the best available evidence and case studies
Source: Implement Sci. 2016 Aug 18;11:117. doi: 10.1186/s13012-016-0472-9 (PMC4990866; doi:10.1186/s13012-016-0472-9)
Supplement: Supplementary file 1 — What are the best strategies to facilitate evidence-informed decision-making: a rapid overview of systematic reviews. (DOCX 323 kb) [file 13012_2016_472_MOESM1_ESM.docx]

# Additional file 1:

# What are the best strategies to facilitate evidence-informed decision making: a rapid overview of systematic reviews

# Abstract

## Background

The work presented in this article is part of a wider body of research aimed at developing methodologies for rapid response for evidence-informed decision making in health policy and practice. This overview sought to answer the question: What are the best strategies to facilitate evidence-informed decision making in health policy and practice?

## Methods

This overview utilized systematic review methods and was conducted according to a pre-defined protocol (PROSPERO registration: CRD42015015998), including clear inclusion criteria. A comprehensive search strategy was used, including published and grey literature, written in English, French, Portuguese or Spanish, from 2004 onwards. Systematic reviews from both developed and developing countries were included. Eleven databases and two websites were searched. Two review authors independently applied the inclusion criteria. Data extraction was done by one reviewer and checked by a second reviewer. The methodological quality of included studies was assessed independently by two reviewers. A narrative summary of the results is presented.

## Results

Fifty-nine systematic reviews met the inclusion criteria for the overview, and data were extracted from 27 systematic reviews of strategy effectiveness. Using the domains of the linking research to action framework, the majority of the interventions that were evaluated in the systematic reviews and found to have a significant impact have focused on push, facilitating pull and pull activities (e.g. dissemination of printed educational materials, including systematic reviews, clinical librarian services, education in evidence-based practice, local opinion leaders, tailored and targeted messaging).  For linkage and exchange – knowledge brokers and interaction between users and producers of research are the only interventions evaluated but the included studies do not provide evidence of effectiveness for these interventions.

## Conclusions

The findings from this research are very useful for guiding action in knowledge translation activities. However, on their own they are insufficient, especially in the policy environment. More and better quality research on interventions to increase the uptake of research into decision-making is needed. A particular area of focus should be on climate as this domain can most affect all others but there is limited research in this area.

# Background

In May 2005 the World Health Assembly called on WHO Member States to “establish or strengthen mechanisms to transfer knowledge in support of evidence-based public health and healthcare delivery systems, and evidence-based health-related policies” [1]. Knowledge translation has been defined by the World Health Organization as: “the synthesis, exchange, and application of knowledge by relevant stakeholders to accelerate the benefits of global and local innovation in strengthening health systems and improving people’s health” [2].

Knowledge translation seeks to address the challenges to the use of scientific evidence in order to close the gap between the evidence generated and decisions made (knowledge translation for action). The term ‘knowledge translation’ has been used interchangeably with the term ‘evidence-informed decision making’ [3], as well as other terms such as ‘knowledge transfer’, ‘knowledge exchange’, ‘research utilization’, and ‘implementation’ [4]. Knowledge translation has also been conceptualized as a term to describe the range of strategies to address the barriers to evidence-informed decision making [5], a concept which was used in this overview.

Different conceptual frameworks^[[1]](#endnote-1)^ have been developed to represent and explain how knowledge translation can, or does, occur [3, 4, 6-10], though none of these frameworks have been tested empirically as a knowledge translation strategy. For the purpose of this overview, the linking research to action framework was adopted to guide the overview, which includes the domains: climate, production of research, push efforts, facilitating pull efforts, pull efforts, linkage and exchange efforts, and evaluation (Table 1) [3, 9].

## Table 1. High level summary of the research to action framework. Adapted from [3, 9]

| **Domain** | **Explanation** |
| --- | --- |
| Climate and context: | Consideration of the local context and climate (i.e. the characteristics, circumstances, and conditions), as well as KT activities. |
| Linkage and Exchange Efforts: | Building relationships between users and researchers. |
| Knowledge Creation: | Creating new knowledge that is timely and relevant. |
| Push Efforts: | Pushing knowledge out to necessary groups in appropriate formats. |
| Facilitating Pull Efforts: | Enabling policymakers to identify relevant research. |
| Pull Efforts: | Pulling the relevant evidence into policy making by the users. |
| Evaluation Efforts | Monitoring and evaluating KT efforts. |

KT: knowledge translation

There are a wide range of knowledge translation strategies available to facilitate the use of research evidence in decision making, with varying degrees of evidence supporting them [11, 12]. Some examples include: a) climate – mandates to support the use of research evidence, e.g. laws, policies; b) push – production of summaries of existing systematic reviews; c) facilitating pull – facilitating access to existing systematic reviews, economic evaluations, clinical practice guidelines, evidence briefs for policy etc.; d) pull – training of research users (policy makers, healthcare practitioners); e) exchange – use of deliberative dialogues informed by evidence briefs; and closer collaboration between researchers and policy-makers.

The overview presented in this article is part of a wider body of research aimed at developing methodologies for rapid response for evidence-informed decision making in health policy and practice based on the best available evidence. The objective of the overview was to use the best available evidence to answer the question: What are the best strategies to facilitate evidence-informed decision making in health policy and practice? We have labelled this study as a rapid overview because it was conducted in a limited timeframe and with the needs of health policy decision-makers in mind.

# Methods

This rapid overview used systematic review methodology and adheres to the Preferred Reporting Items for Systematic Reviews and Meta-Analysis (PRISMA) statement [13]. A systematic review protocol was written and registered prior to undertaking the searches [14]. Deviations from the protocol are listed in Additional file 2 (see: Changes to the protocol).

## Inclusion criteria for studies

Studies were selected based on the following inclusion criteria:

**Types of studies:** Only systematic reviews were included as this is a very well-researched area and preliminary searches revealed the existence of many relevant systematic reviews. We did not use an a priori definition of a systematic review but considered reviews that described their methods, including sources of studies, search terms and inclusion criteria as systematic reviews.

**Types of participants:** Apart from needing to be within the field of health policy and practice, the types of participants were not restricted, and the level of analysis could be at the level of the individual, organization, system or geographical area.

**Types of articles/interventions:** Articles that evaluated knowledge translation strategies for health policy and/or practice were included. Articles that evaluated barriers and/or facilitators to the uptake of research evidence were also sought in case sufficient evidence of strategy effectiveness could not be found.

**Types of comparisons:** Suitable comparisons (where relevant to the article type) included: no intervention, another intervention, or current practice.

**Types of outcome measures:** Relevant outcome measures included: measures of research uptake / use; change in knowledge, skill or practice related to the uptake of research (as defined by LaRocca and colleagues [15]); changes in organizational structures or policy to support the use of research; organizational-level change in how research can be or is used; changes in system-level structures or policy to support the use of research; system-level change in how research can be or is used; effective implementation; and cost-effectiveness.

Publications in English, French, Portuguese or Spanish, from any country and published from 2004 onwards were included. The year 2004 was chosen as this is the year of the Mexico Ministerial Summit on Health Research, where the know-do gap was first given serious attention by health ministers [16]. Both grey and peer-reviewed literature was sought and included.

For systematic reviews of clinical practice guideline (CPG) implementation and clinical decision support systems (CDSS) implementation, systematic reviews were only included if the systematic review specifically noted in the inclusion criteria that the CPG or CDSS had to be based on research evidence. This decision was made during the study selection process because not all CPGs or CDSSs are based on research evidence [17, 18]. This criterion has also been used in other systematic reviews [19]. This assumes that 'guideline use' can be considered 'research use' provided it is explicitly 'evidence-based'.

## Search methods for identification of studies

A comprehensive search of eleven databases and two websites was conducted. The databases searched were: PubMed, EMBASE, CINAHL, LILACS, Health Systems Evidence, The Cochrane Library (including Cochrane Reviews, the Database of Abstracts of Reviews of Effectiveness, the Health Technology Assessment database, NHS Economic Evaluation Database, and the database of Methods Studies), and EconLit. The websites searched were Google and Google Scholar.

### Grey literature and manual search

Some of the selected databases index a combination of published and unpublished studies (for example, doctoral dissertations, conference abstracts and unpublished reports) therefore unpublished studies were partially captured through the electronic search process. In addition Google and Google Scholar were searched. Some of the authors’ own databases of knowledge translation literature were also searched by hand for relevant studies. The reference list of included studies was searched. Contact was made with 20 key authors and experts in the area for further studies, of whom 14 responded. Finally, due to a perception by one of the authors (JL) that there were systematic reviews missing from the search for knowledge translation to practice a supplementary targeted search of the Health Systems Evidence database was conducted. This was done by using the advanced search option of ‘Topic’ and searching for recent high quality systematic reviews within each relevant category.

## Table 2 – Keyword areas for searching

| **Keyword Areas** | **Details** |
| --- | --- |
| Knowledge translation | “research uptake” OR “research use” OR “use of research” OR “evidence informed” OR “evidence-informed” OR “decision making” OR “decision-making” OR “research utilization” OR “research utilisation” OR “technology transfer” OR “knowledge-to-action” OR “knowledge to action” OR “implementation science” OR “information dissemination” OR “diffusion of innovation” OR “knowledge generation” OR “knowledge translation” OR “knowledge transfer” OR “knowledge uptake” OR “knowledge exchange” OR “knowledge broker*” OR “knowledge mobilization” OR “knowledge mobilisation” |
| Systematic reviews | “systematic review” OR "meta-analysis" OR MEDLINE |

### Search strategy

Searches were conducted between 21^st^ January and 18^th^ February 2015 and supplementary searches (reference lists, contact with authors, and targeted search of Health Systems Evidence) were conducted in May 2015. Databases were searched using keywords from both keyword areas – combined using ‘AND’ (Table 2). Keywords were searched for in the title and abstract fields, except where otherwise stated in Additional file 2 (see: Search terms). Results were downloaded into the EndNote reference management program (version X7) and duplicates removed. The internet search utilized the search terms: ‘information dissemination’; ‘knowledge translation’; ‘research use’; and ‘research uptake’ – combined with ‘systematic review’.

## Screening and selection of studies

Searches were conducted and titles and abstracts screened according to the selection criteria by one review author (MH). The full text of any potentially relevant papers was retrieved for closer examination. This reviewer erred on the side of inclusion where there was any doubt about its inclusion to ensure any potentially relevant papers were not missed. The inclusion criteria were then applied against the full text version of the papers (where available) independently by two reviewers (MH and RC). For studies in Portuguese and Spanish, EC, LR or JB played the role of second reviewer. Disagreements regarding eligibility of studies were resolved by discussion and consensus. Where the two reviewers were still uncertain about inclusion, the other reviewers (EC, LR, JB, JL) were asked to provide input and consensus reached. All studies which initially appeared to meet the inclusion criteria but on inspection of the full text paper did not meet the inclusion criteria were detailed in a table ‘Characteristics of excluded systematic reviews’ together with reasons for their exclusion.

All systematic reviews of a) strategy effectiveness and/or b) barriers and facilitators to the uptake of research evidence that met the inclusion criteria were selected. At this stage the systematic review was categorized as a) strategy effectiveness and/or b) barriers and facilitators. The systematic review was also assessed for its scope or application to: policy, practice, or both policy and practice. When classifying papers related to policy we took an expansive view of policy and thus included local level and institutional policy decisions [20]. The reason for classifying systematic reviews was so to enable the reviewers to make a decision as to which systematic reviews should proceed to data extraction as it was clear that there were many potentially relevant systematic reviews and resources may not be sufficient to analyze all of them in time to meet the funders’ deadlines. Following discussion between all co-authors it was decided to limit data extraction to systematic reviews of strategy effectiveness but to include both policy and practice.

## Data extraction

Systematic reviews that were specific to a single health issue (e.g. pain management) were excluded from data extraction in order to focus on the systematic reviews that were generalizable across health care issues. In the same manner, systematic reviews specific to a single profession (e.g. nursing, allied health, rehabilitation clinicians) were excluded from data extraction, except where no other systematic review addressed the particular intervention across professions. Further, where there were multiple systematic reviews addressing the same intervention or question, data were only extracted from the most recent good quality review(s). This was done to reduce the risk of double-counting of the same primary studies, while maximizing the quality of the evidence.

Information extracted from studies included: objectives, target population, setting, strategy tested, outcomes reported, date of last search, included study designs, country of studies, and results. Data extraction was done by one reviewer (MH) and checked by a second reviewer (RC). Disagreements were resolved through discussion and consensus.

When extracting data from the included systematic reviews we allocated each strategy to a domain from Lavis’ and colleague’s framework to assess country-level efforts to link research to action (Table 1). When classifying individual interventions we considered: 1) Who were the main players – producers or purveyors of research, and/or research users (healthcare professionals, civil servants); 2) The content and intent of the intervention; and 3) The elements provided by Lavis and colleagues in their framework (see Table 1 in: [9]). Classification of an intervention was done by one reviewer (MH) and checked by a second reviewer (RC).

## Assessment of methodological quality

The methodological quality of included systematic reviews was assessed independently by two reviewers using AMSTAR: A MeaSurement Tool to Assess Reviews [21]. Disagreements in scoring were resolved by discussion and consensus. For this overview, systematic reviews that achieved AMSTAR scores of 8 to 11 were considered high quality; scores of 4 to 7 medium quality; and scores of 0 to 3 low quality. These cut-offs are commonly used in Cochrane Collaboration overviews. The study quality assessment was used to interpret the results during the synthesis phase and in the formulation of conclusions.

## Data analysis

Findings from the included publications were synthesized using tables and a narrative summary. Meta-analysis was not possible because the included studies were heterogeneous in terms of the populations, interventions and outcomes tested.

# Results

### Search results

59 systematic reviews (from 62 articles) met the inclusion criteria for the overview. Forty systematic reviews (from 43 articles) addressed the effectiveness of knowledge translation strategies and 26 systematic reviews (from 27 articles) addressed barriers and facilitators to the uptake of research evidence (Note that these numbers do not add up to 59 as some systematic reviews addressed both strategy effectiveness and barriers and facilitators). Data were extracted from 27 systematic reviews (29 articles) of strategy effectiveness [7, 12, 15, 22-47]. The remaining 13 systematic reviews (14 articles) addressing effectiveness of knowledge translation strategies that met the inclusion criteria but for which data were not extracted are referenced in Additional file 2 - Table 1, along with the reason for their exclusion from data extraction. References for the 26 systematic reviews of barriers and facilitators that were excluded from data extraction (for the barriers and facilitators component only) are also listed in Additional file 2.

The selection process for studies and the numbers at each stage are shown in Figure 1. The reasons for exclusion of the 78 (62 + 16) papers at full text stage are shown in Additional file 2 – Table 2. The additional searches identified an additional 27 potential articles of which 11 met the inclusion criteria - four came from searches of the reference list of included studies, seven from contact with authors, and none from the additional manual searching of the Health Systems Evidence database.

### Characteristics of included studies and quality assessment

Details of the characteristics of the included systematic reviews for which data were extracted are in Additional file 2 – Tables 3a-3c (n=27). Fifteen of the systematic reviews were applicable to practice only, five to policy only, and seven to policy and practice. Few primary studies were conducted in developing countries.

AMSTAR scores ranged from 2 to 10 out of a possible maximum of 11, with 11 systematic reviews being of high quality with scores between 8 and 11; 11 being of medium quality with scores between 4 and 7; and 4 low quality with scores between 0 and 3. Only four of the included systematic reviews stated whether the primary studies may be impacted by conflicts of interest. The AMSTAR quality assessment is shown in Tables 3-5 (with full details in Additional file 2 – Tables 4-6). The findings of low quality systematic reviews should be treated with caution [7, 12, 33, 34, 37, 39].

### Findings

The knowledge translation strategies tested in the 27 included systematic reviews for which data were extracted, and the main findings from these systematic reviews are shown in Tables 3-5. A wide variety of strategies were tested, with the majority of strategies being applicable to practice only, which is likely due to the larger number of primary studies conducted with healthcare practitioners than with policymakers.

It is important to clarify here that, while the systematic review may be applicable to both policy and practice, not all strategies tested in the included primary studies may have been tested with both policymakers and practitioners. For example, the systematic review by Wallace and colleagues is applicable to both policy and practice but the strategy of disseminating short summaries of systematic reviews that was included in this review was only tested in one RCT that was conducted with practitioners [45]. Thus, the strategy is categorized as ‘practice only’ in Table 5, while the systematic review is categorized as ‘policy & practice’ in Additional file 2 – Table 3b.

Three systematic reviews are missing from Tables 3-5 but are included in Additional file 2. One of these was presented at an international conference but has never been published as a full report – thus, the findings were not usable and an AMSTAR assessment could not be conducted [46]. The second did not make any conclusions about specific interventions due to the poor quality of included primary studies [41]. The third was focused on research use in health policy in low and middle-income countries, with most of the included studies being descriptive case studies [26]. Thus, the authors of the review did not draw any conclusions about specific interventions. They did note, however, that the findings were broadly consistent with the findings from high-income countries on the need for multi-faceted, tailored interventions and on the importance of contextual influences, particularly organizational [26].

Strategies for which there is some evidence of effectiveness on some outcomes for practice, policy or both are shown in Table 6. This summary is based on the results in Table 3-5 and Additional file 2 – Tables 3a-3c. No studies were found on the impact of rapid reviews on evidence-informed decision making.

# Discussion

In regards to successful knowledge translation strategies, the majority of the interventions that were evaluated in the included systematic reviews and found to have a significant impact have focused on ‘push’, ‘facilitating pull’ and ‘pull’ activities (Table 6). Strategies for which there is evidence of effectiveness on at least one outcome in the practice environment include: access to clinical information retrieval technology (e.g. Medline) [30, 38], clinical librarian services [23], education in evidence-based practice [27, 36] or critical appraisal [35], educational outreach when combined with access to the Cochrane Library and other materials [44, 45], dissemination of printed educational materials [32], including short summaries of systematic reviews [45], local opinion leaders [28] and some toolkits [47]. In the policy environment successful strategies included: tailored and targeted messaging [48], disseminating evidence [15], including systematic reviews [43], and training in the appraisal and use of research [12].  In the policy and practice environments, successful strategies include: mass mailing a printed bulletin which summarizes systematic review evidence [42], and one multifaceted intervention [15]. Toolkits were the only intervention to potentially impact on ‘climate’. For linkage and exchange – knowledge brokers and interaction between users and producers of research are the only interventions evaluated but the included studies did not find an effect of these interventions [12, 48].

Some interventions have been shown in previous systematic reviews to be effective for improving clinical practice but are not included in our list of effective strategies for evidence-informed decision making [11, 49]. Examples include audit and feedback, educational outreach and computerized reminders. The most likely reason for this is our deliberate focus on uptake of research evidence and exclusion of systematic reviews that were not specifically focused on research use. This was a particular issue for clinical decision support systems (CDSSs) and clinical practice guidelines (CPGs), but also for audit and feedback, computerized reminders, and educational outreach, where the most recent systematic reviews were excluded from this overview [50-52] or not picked up by our search strategy [53]. While it could be argued that our search strategy was the problem, as we did not specify individual interventions by name, the fact that the additional manual search of the Health Systems Evidence database by intervention category did not find further relevant reviews suggests that this is not the case.

Regarding the quality of the included systematic reviews, on average, the systematic reviews of interventions applicable to healthcare practitioners were of higher quality (average score 7.2 for the practice only systematic reviews) than those applicable to policy makers (average score 5.4 for the policy only systematic reviews). This is likely to be due to the longer history of conducting systematic reviews in the clinical field and the greater acceptance and ease of conducting RCTs than in the policy arena. It is also important to note that the supporting evidence for a particular intervention, though cited in a systematic review, could be limited to a single study. This was a particular issue for interventions in the policy environment. We have attempted to highlight this issue by stating in Table 6 where the evidence is based on one study.

## Strengths and limitations

A key strength of this overview is the use of high quality systematic review methodology, including the consideration of the scientific quality of the included studies in formulating conclusions. A meta-analysis was not possible due to the heterogeneity in terms of intervention types and populations studied in the included systematic reviews. As a result publication bias could not be assessed quantitatively in this overview and no clear methods are available for assessing publication bias qualitatively [54]. Shortcuts taken to make this review more rapid, as well as an AMSTAR assessment of the review, are shown in Additional file 2 – Shortcuts taken.

A second strength of this overview is that we limited study selection to interventions explicitly focused on improving the uptake of research. This resulted in the exclusion of several systematic reviews of CPG and CDSS implementation and various systematic reviews of interventions aimed at practice improvement (e.g. audit and feedback, reminders and prompts). This decision is justified as not all CPGs, CDSSs, and practices promoted in clinical care are explicitly based on research evidence [17, 18, 55].

A third strength of this overview is that we deliberately tried to reduce the risk of double-counting of the same primary studies, which can bias the results towards primary studies that have been counted in multiple overviews [56]. We did this by examining the included references in each systematic review to highlight primary studies included in more than one systematic review. This was a particular issue for one RCT that has been included in multiple systematic reviews included in the overview [48]. When presenting the results of the trial we noted the systematic reviews in which it was included but attributed the results to the RCT, not the systematic reviews.

Possible limitations of this overview include: 1) The exclusion from data extraction of 13 systematic reviews that were specific to a single health issue or to a single profession; and some older systematic reviews that addressed the same intervention or question as more recent good quality review(s). However, this was done to reduce the risk of double-counting of the same primary studies; 2) Exclusion of systematic reviews on barriers and facilitators from data extraction due to time limitations; 3) The fact that few primary studies were conducted in developing countries, which is an issue for the generalizability of the results; 4) The interventions that could be included are limited to those for which a systematic review was conducted. However, this is a very extensively researched area and it is unlikely that interventions tested in good quality primary studies would have been missed unless they were published in the last few years; 5) Restricting the search to articles in English, French, Portuguese or Spanish (languages with which the review authors are competent) and to 2004 onwards. However, it was necessary to expedite the review process to ensure its results could be used in decision-making and is unlikely to have resulted in the loss of important evidence.

## Implications for research

While interventions for knowledge translation have been widely researched the greatest focus to date has been on the practice rather than the policy environments and, within the former, on push, facilitating pull, and pull activities. More and better quality research of interventions to increase the uptake of research into policy is needed. In both the policy and practice environments further research is needed on interventions that impact on the domains of: climate; production of research; or evaluation of knowledge translation efforts. For linkage and exchange activities, knowledge brokering requires further testing using an intervention with greater reach and intensity, as do other interventions in this domain. There are also a number of interventions (listed in Tables 3-5) for which research was limited or lacking. These include: educational meetings; journal clubs; communities of practice; mentoring; interaction between users and producers of research; access to systematic reviews (policy makers); knowledge brokering; packaging of systematic reviews as summaries, overviews or policy briefs; external inspection of compliance with standards; managerial leadership for nurses; and several multifaceted interventions.

When designing and testing new interventions to facilitate the uptake of research, known barriers and facilitators need to be considered. The 26 systematic reviews found on this topic will be important in guiding this process. It is also important to be mindful of the realities of policy decision-making where other issues such as context, public opinion, acceptability to stakeholders, and feasibility of implementation are also important considerations [57, 58].

The biggest, and perhaps most important, research gap in knowledge translation research is in the area of climate as it is this domain that can most affect all others. For example, if funders have a mandate to link research to action, all other interventions will have a much greater chance of success. In addition, in the linkage and exchange domain, the development of partnerships is seen as important to overcome the gap between research and policy [9, 59] but needs to be tested as do deliberative dialogues [60, 61].

## Implications for policy and practice

The findings from this research will be useful for guiding action in knowledge translation activities for both policy and practice. However, on their own are insufficient, especially in the policy environment. Users of research, such as civil servants and healthcare professionals, should support further research in this area. Research users should also consider additional actions to increase the uptake of research, especially in the climate and linkage and exchange domains. At the same time they should ensure that these actions undergo a proper evaluation to contribute to the evidence base. Interventions that could be considered for testing include: employing more researchers in policy positions; joint appointments of researchers and policymakers across policy and academic institutions; and rewards and/or recognition for academics and policymakers who work towards linking research to action – which are all interventions that fit within the climate domain of the linking research to action framework [9] and could also facilitate the development of partnerships (linkage and exchange).

# Conclusions

The majority of the interventions that were evaluated in the included systematic reviews and found to have a significant impact have focused on push, pull and facilitating pull activities.  The findings from this research will be very useful for guiding action in knowledge translation activities. However, they need to be supplemented with more and better quality research in the areas of climate, production of research, linkage and exchange, and evaluation of knowledge translation efforts. A particular area of focus should be on climate as this domain can most affect all others. A greater focus is also needed on high quality trials in the policy environment.

# Competing interests

The author(s) declare that they have no competing interests. Neither the Ministry of Health of Brazil nor the Pan American Health Organization (PAHO) have a vested interest in any of the interventions included in this overview – though they do have a professional interest in increasing the uptake of research evidence in decision making, as do MH, RC, LR and MH. Two of the review authors (EC and LR) are employees of PAHO and one was an employee of the Ministry of Health of Brazil at the time of the study (JB). However, the views and opinions expressed herein are those of the overview authors and do not necessarily reflect the views of the Ministry of Health of Brazil or the Pan American Health Organization.

# Authors' contributions

EC and JB had the original idea for the overview and obtained funding; MH and EC wrote the protocol with input from RC, JB, and LR; JL provided extensive input to methods and decision-making during the study selection and data extraction process; MH and RC undertook the study selection, data extraction and quality assessment; MH undertook data synthesis and drafted the manuscript; JL, EC, RC, JB and LR provided guidance throughout the selection, data extraction and synthesis phase of the overview; all authors provided commentary on and approved the final manuscript.

# Acknowledgements

We thank the authors of included studies and other experts in the field who responded to our request to identify further studies that could meet our inclusion criteria.

## Funding

This work was developed and funded under the cooperation agreement # 47 between the Department of Science and Technology of the Ministry of Health of Brazil and the Pan American Health Organization. The funders of this study set the terms of reference for the project but, apart from the input of JB, EC and LR to the conduct of the study did not significantly influence the work. Manuscript preparation was funded by the Ministry of Health Brazil, through an EVIPNet Brazil project with the Bireme/PAHO.

# Endnotes

# References

1. World Health Assembly. Resolution on health research. Available from: <http://www.who.int/rpc/meetings/58th_WHA_resolution.pdf> 2005.

2. WHO. Bridging the “Know–Do” Gap: Meeting on Knowledge Translation in Global Health, 10–12 October 2005. World Health Organization, 2006 Contract No.: WHO/EIP/KMS/2006.2.

3. WHO. Knowledge Translation Framework for Ageing and Health. Geneva: Department of Ageing and Life-Course, World Health Organization, 2012.

4. Graham ID, Logan J, Harrison MB, Straus SE, Tetroe J, Caswell W, et al. Lost in knowledge translation: time for a map? J Contin Educ Health Prof. 2006;26:13-24.

5. Armstrong R, Waters E, Dobbins M, Anderson L, Moore L, Petticrew M, et al. Knowledge translation strategies to improve the use of evidence in public health decision making in local government: intervention design and implementation plan. Implement Sci. 2013;8:121.

6. Best A, Holmes B. Systems thinking, knowledge and action: towards better models and methods. Evidence & Policy: A Journal of Research, Debate and Practice. 2010;6:145-59.

7. Moore G, Todd A, Redman S. Strategies to increase the use of evidence from research in population health policy and programs: Evidence Check rapid reviews brokered by the Sax Institute (<http://www.saxinstitute.org.au>) for the Primary Health and Community Partnerships Unit, NSW Department of Health. 2009.

8. Greenhalgh T, Robert G, Macfarlane F, Bate P, Kyriakidou O. Diffusion of innovations in service organizations: systematic review and recommendations. Milbank Q. 2004;82:581-629.

9. Lavis JN, Lomas J, Hamid M, Sewankambo NK. Assessing country-level efforts to link research to action. Bull World Health Organ. 2006;84:620-8.

10. Redman S, Jorm L, Haines M. Increasing the use of research in health policy : the Sax Institute model. Australasian Epidemiologist. 2008;15:15-8.

11. Grimshaw JM, Eccles MP, Lavis JN, Hill SJ, Squires JE. Knowledge translation of research findings. Implement Sci. 2012;7:50.

12. Moore G, Redman S, Haines M, Todd A. What works to increase the use of research in population health policy and programmes: A review. Evidence & Policy. 2011;7:277-305.

13. Moher D, Liberati A, Tetzlaff J, Altman DG, Group P. Preferred reporting items for systematic reviews and meta-analyses: the PRISMA statement. PLoS Med. 2009;6:e1000097.

14. Haby M, Chapman E, Reveiz L, Barreto J, Clark R. Methodologies for rapid response for evidence-informed decision making in health policy and practice: an overview of systematic reviews and primary studies (Protocol). PROSPERO: CRD42015015998. 2015.

15. LaRocca R, Yost J, Dobbins M, Ciliska D, Butt M. The effectiveness of knowledge translation strategies used in public health: a systematic review. BMC Public Health. 2012;12:751.

16. WHO. Report from the Ministerial Summit on Health Research: identify challenges, inform actions, correct inequities. Mexico City, 16-20 November 2004. Available at: <http://www.who.int/rpc/summit>. World Health Organization, 2005 Nov. Report No.

17. De Luca MJF, Esandi ME, Guzman J, Reveiz L. Strategies for implementation of clinical practice guidelines in Latin America and the Caribbean: a systematic review. PLoS One. 2015;(to be submitted).

18. Hemens BJ, Holbrook A, Tonkin M, Mackay JA, Weise-Kelly L, Navarro T, et al. Computerized clinical decision support systems for drug prescribing and management: A decision-maker-researcher partnership systematic review. Implement Sci. 2011;6:89.

19. Ospina MB, Taenzer P, Rashiq S, MacDermid JC, Carr E, Chojecki D, et al. A systematic review of the effectiveness of knowledge translation interventions for chronic noncancer pain management. Pain Research & Management. 2013;18:e129-41.

20. Seavey J, McGrath R, Aytur S. Health policy analysis: framework and tools for success. New York, NY: Springer; 2014.

21. Shea BJ, Grimshaw JM, Wells GA, Boers M, Andersson N, Hamel C, et al. Development of AMSTAR: a measurement tool to assess the methodological quality of systematic reviews. BMC Med Res Methodol. 2007;7:10.

22. Abdullah G, Rossy D, Ploeg J, Davies B, Higuchi K, Sikora L, et al. Measuring the effectiveness of mentoring as a knowledge translation intervention for implementing empirical evidence: a systematic review. Worldviews Evid Based Nurs. 2014;11:284-300.

23. Brettle A, Maden-Jenkins M, Anderson L, McNally R, Pratchett T, Tancock J, et al. Evaluating clinical librarian services: a systematic review. Health Information & Libraries Journal. 2011;28:3-22.

24. Bunn F, Sworn K. Strategies to promote the impact of systematic reviews on healthcare policy: A systematic review of the literature. Evidence & Policy. 2011;7:403-28.

25. Chambers D, Wilson PM, Thompson CA, Hanbury A, Farley K, Light K. Maximizing the impact of systematic reviews in health care decision making: a systematic scoping review of knowledge-translation resources. Milbank Q. 2011;89:131-56.

26. Clar C, Campbell S, Davidson L, Graham W. What are the effects of interventions to improve the uptake of evidence from health research into policy in low and middle-income countries? Final Report to DFID. Ref.No. (PO) 40032846. London, UK: Research for Development (R4D), Department for International Development, 2011.

27. Dizon JM, Grimmer-Somers KA, Kumar S. Current evidence on evidence-based practice training in allied health: a systematic review of the literature. International Journal of Evidence-Based Healthcare. 2012;10:347-60.

28. Flodgren G, Parmelli E, Doumit G, Gattellari M, O'Brien MA, Grimshaw J, et al. Local opinion leaders: effects on professional practice and health care outcomes. Cochrane Database Syst Rev. 2011;Issue 8:Art. No. CD000125.

29. Flodgren G, Pomey MP, Taber SA, Eccles MP. Effectiveness of external inspection of compliance with standards in improving healthcare organisation behaviour, healthcare professional behaviour or patient outcomes. Cochrane Database Syst Rev. 2011:CD008992.

30. Gagnon MP, Pluye P, Desmartis M, Car J, Pagliari C, Labrecque M, et al. A systematic review of interventions promoting clinical information retrieval technology (CIRT) adoption by healthcare professionals. Int J Med Inf. 2010;79:669-80.

31. Gifford W, Davies B, Edwards N, Griffin P, Lybanon V. Managerial leadership for nurses' use of research evidence: an integrative review of the literature. Worldviews Evid Based Nurs. 2007;4:126-45.

32. Giguere A, Legare F, Grimshaw J, Turcotte S, Fiander M, Grudniewicz A, et al. Printed educational materials: effects on professional practice and healthcare outcomes. Cochrane Database Syst Rev. 2012;10:CD004398.

33. Harris J, Kearley K, Heneghan C, Meats E, Kearley-Shiers K, Roberts N, et al. Are journal clubs effective in supporting evidence-based decision making? A systematic review. BEME [Internet]. 2008. Available from: <http://onlinelibrary.wiley.com/o/cochrane/cldare/articles/DARE-12011000676/frame.html>.

34. Harris J, Kearley K, Heneghan C, Meats E, Roberts N, Perera R, et al. Are journal clubs effective in supporting evidence-based decision making? A systematic review. BEME Guide No. 16. Med Teach. 2011;33:9-23.

35. Horsley T, Hyde C, Santesso N, Parkes J, Milne R, Stewart R. Teaching critical appraisal skills in healthcare settings. Cochrane Database Syst Rev. 2011:CD001270.

36. Ilic D, Maloney S. Methods of teaching medical trainees evidence-based medicine: a systematic review. Med Educ. 2014;48:124-35.

37. Li L, Grimshaw J, Nielsen C, Judd M, Coyte P, Graham I. Use of communities of practice in business and health care sectors: A systematic review. Implement Sci. 2009;4:27.

38. Lobach D, Sanders GD, Bright TJ, Wong A, Dhurjati R, Bristow E, et al. Enabling health care decisionmaking through clinical decision support and knowledge management. Evidence Report No. 203. (Prepared by the Duke Evidence-based Practice Center under Contract No. 290-2007-10066-I.) AHRQ Publication No. 12-E001-EF2012. Available from: <http://onlinelibrary.wiley.com/o/cochrane/clhta/articles/HTA-32012000924/frame.html>.

39. McCormack B, Rycroft-Malone J, DeCorby K, Hutchinson AM, Bucknall T, Kent B, et al. A realist review of interventions and strategies to promote evidence-informed healthcare: A focus on change agency. Implement Sci. 2013;8:107.

40. McCormack L, Sheridan S, Lewis M, Boudewyns V, Melvin CL, Kistler C, et al. Communication and dissemination strategies to facilitate the use of health-related evidence. Evidence Report/Technology Assessment No. 213. (Prepared by the RTI International–University of North Carolina Evidence-based Practice Center under Contract No. 290-2007-10056-I.) AHRQ Publication No. 13(14)-E003-EF. Rockville, MD: Agency for Healthcare Research and Quality, 2013.

41. Mitton C, Adair CE, McKenzie E, Patten SB, Waye Perry B. Knowledge transfer and exchange: review and synthesis of the literature. Milbank Quarterly. 2007;85:729-68.

42. Murthy L, Shepperd S, Clarke MJ, Garner SE, Lavis JN, Perrier L, et al. Interventions to improve the use of systematic reviews in decision-making by health system managers, policy makers and clinicians. Cochrane Database Syst Rev. 2012;Issue 9:Art. No. CD009401.

43. Perrier L, Mrklas K, Lavis JN, Straus SE. Interventions encouraging the use of systematic reviews by health policymakers and managers: a systematic review. Implement Sci. 2011;6:43.

44. Perrier L, Mrklas K, Shepperd S, Dobbins M, McKibbon KA, Straus SE. Interventions encouraging the use of systematic reviews in clinical decision-making: a systematic review. J Gen Intern Med. 2011;26:419-26.

45. Wallace J, Byrne C, Clarke M. Improving the uptake of systematic reviews: a systematic review of intervention effectiveness and relevance. BMJ Open. 2014;4:e005834.

46. Worbes-Cerezo M, Linertova R, Serrano-Aguilar P. Cost-effectiveness of knowledge translation methods [Abstract]. Value Health. 2010;13:A421.

47. Yamada J, Shorkey A, Barwick M, Widger K, Stevens BJ. The effectiveness of toolkits as knowledge translation strategies for integrating evidence into clinical care: a systematic review. BMJ Open. 2015;5:e006808.

48. Dobbins M, Hanna SE, Ciliska D, Manske S, Cameron R, Mercer SL, et al. A randomized controlled trial evaluating the impact of knowledge translation and exchange strategies. Implement Sci. 2009;4:61.

49. Boaz A, Baeza J, Fraser A, European Implementation Score Collaborative G. Effective implementation of research into practice: an overview of systematic reviews of the health literature. BMC Res Notes. 2011;4:212.

50. Ivers N, Jamtvedt G, Flottorp S, Young JM, Odgaard-Jensen J, French SD, et al. Audit and feedback: effects on professional practice and healthcare outcomes. Cochrane Database Syst Rev. 2012;6:CD000259.

51. Arditi C, Rege-Walther M, Wyatt JC, Durieux P, Burnand B. Computer-generated reminders delivered on paper to healthcare professionals; effects on professional practice and health care outcomes. Cochrane Database Syst Rev. 2012;12:CD001175.

52. Holt TA, Thorogood M, Griffiths F. Changing clinical practice through patient specific reminders available at the time of the clinical encounter: systematic review and meta-analysis. J Gen Intern Med. 2012;27:974-84.

53. O'Brien MA, Rogers S, Jamtvedt G, Oxman AD, Odgaard-Jensen J, Kristoffersen DT, et al. Educational outreach visits: effects on professional practice and health care outcomes. Cochrane Database of Systematic Reviews. 2007;Issue 4:CD000409.

54. Song F, Parekh S, Hooper L, Loke YK, Ryder J. Dissemination and publication of research findings: an updated review of related biases. Health Technol Assess. 2010;14 (8).

55. Chalmers I. If evidence-informed policy works in practice, does it matter if it doesn't work in theory? Evidence & Policy: A Journal of Research, Debate and Practice. 2005;1:227-42.

56. Pieper D, Antoine SL, Mathes T, Neugebauer EA, Eikermann M. Systematic review finds overlapping reviews were not mentioned in every other overview. Journal of Clinical Epidemiology. 2014;67:368-75.

57. Clark R, Haby M. Chapter 7. Evidence-based policy: why and how. In: Carey G, Landvogt K, Barraket J, editors. Creating and implementing public policy: Cross-sectoral debates. Routledge, Taylor and Francis Group. London: Policy Press; 2015.

58. Colebatch HK. Policy. 2nd ed. Buckingham: Open University Press; 2002.

59. Moodie R. Where different worlds collide: expanding the influence of research and researchers on policy. J Public Health Policy. 2009;30(Suppl 1):S33-S7.

60. Boyko JA, Lavis JN, Abelson J, Dobbins M, Carter N. Deliberative dialogues as a mechanism for knowledge translation and exchange in health systems decision-making. Soc Sci Med. 2012;75:1938-45.

61. Moat KA, Lavis JN, Clancy SJ, El-Jardali F, Pantoja T, Knowledge Translation Platform Evaluation study team. Evidence briefs and deliberative dialogues: perceptions and intentions to act on what was learnt. Bull World Health Organ. 2014;92:20-8.

## Figure 1 - Study selection flow chart – Knowledge translation strategies

Full-text articles excluded, with reasons
n = 16

Additional articles identified through supplementary searching

n = 27

Systematic reviews of strategy effectiveness

n = 40 (from 43 articles)

Systematic reviews that met the inclusion criteria

n = 59 (from 62 articles)

Systematic reviews of barriers and facilitators

n = 26 (from 27 articles)

Records excluded
n = 3116

Duplicates removed
n = 724

Records identified through database searching
n = 3940

Additional records identified through other sources
n = 13

Total records identified
n = 3953

Records screened
(titles and abstracts)
n = 3229

Full-text articles excluded, with reasons
n = 62

Full-text articles assessed for eligibility
n = 113

EEs: n=10

Systematic reviews that met the inclusion criteria

n = 48 (from 51 articles)

Systematic reviews that proceeded to data extraction

n = 27 (from 29 articles)

## Table 3 - Strategies tested in the practice environment only, quality scores, and main findings of the included systematic reviews

| Strategy | Domain | Systematic review/s* and AMSTAR score | Findings |
| --- | --- | --- | --- |
| Access to clinical information retrieval technology (CIRT) / knowledge management systems (e.g. Medline) | Pull Efforts | Gagnon [30] – 5  Lobach [38] – 10 | Overall, CIRT capability (defined as the proportion of health care professionals capable of performing a search, successfully searching, or using CIRT) improved with intervention (OR=2.10; 95% CI; 1.63 to 2.71; p=0.0001), though there was significant heterogeneity in the studies [30]. Impact on clinical outcomes, health care process measures, workload and efficiency for the user, or organization of health care delivery outcomes is not known [38]. |
| Clinical librarian services | Facilitating Pull Efforts | Brettle [23] – 8 | Clinical librarian services are effective in providing relevant and useful information and are perceived to save clinicians’ time. The majority of studies reported a positive impact on patient care; a quarter of studies identified a positive impact on the choice of drug or therapy. On the whole, however only a small number of studies were able or sought to quantify the impact made by the clinical librarian [23]. |
| Education in evidence-based practice (EBP) | Pull Efforts | Dizon [27] – 6  Ilic [36] – 6 | For allied health professionals – knowledge and skills were influenced by any EBP training program [27]. EBP programs that utilised co-interventions such as opinion leaders resulted in improvements in attitudes [27]. For medical trainees (undergraduate or postgraduate) – learner competency in EBM (knowledge, skills, attitudes or behavior towards EBM) increased post-intervention across all studies [36]. However, it cannot be concluded that education in EBM improves competency in EBM – only two of the RCTs had a true control group and results were mixed. No difference in learner outcomes was identified across a variety of educational modes [36]. |
| Education in critical appraisal | Pull Efforts | Horsley [35] – 10 | Low-intensity critical appraisal teaching interventions in healthcare populations may result in modest gains in knowledge [35]. |
| Education, educational visits (outreach) | Pull Efforts | LaRocca [15] – 7  Perrier [44] – 9  Wallace [45] – 8 | The educational session was insufficient to significantly change adherence to a national suicide prevention guideline for healthcare practitioners [15]. A single informal educational visit by an obstetrician plus donation of the Cochrane database and other materials significantly improved one clinical practice (out of four clinical practices measured) for healthcare practitioners – evidence from one RCT reported in two systematic reviews [44, 45]. |
| Dissemination of printed educational materials (PEMs) | Push Efforts | Giguere [32] – 7 | The results of this review suggest that when used alone and compared to no intervention, PEMs may have a small beneficial effect on professional practice outcomes [32]. There is insufficient information to reliably estimate the effect of PEMs on patient outcomes [32]. |
| Disseminating short summaries of systematic reviews | Push Efforts | Wallace [45] – 8 | One RCT found that four short, one-page systematic review summaries delivered by email or mail to nurses, on patient-controlled analgesia improve awareness of review evidence [45]. |
| Single vs multicomponent dissemination strategies | Push Efforts | McCormack [40] – 9 | For disseminating evidence, compared with single dissemination strategies, multicomponent dissemination strategies were more effective at enhancing clinician behavior, particularly for guideline adherence [40]. |
| Communicating evidence, communicating uncertainty | Push Efforts | McCormack [40] – 9 | For communicating evidence, investigators frequently blend more than one communication strategy in interventions and the evidence was insufficient to make recommendations [40]. Key findings for communicating uncertainty indicate that evidence on communicating overall strength of recommendation and precision was insufficient, but certain ways of communicating directness and net benefit may be helpful in reducing uncertainty [40]. |
| Journal clubs | Pull Efforts | Harris et al. 2011 [34] – 3 | The effectiveness of journal clubs in supporting evidence-based decision making is not clear because only seven studies attempted to measure this endpoint and they relied on self-report. Studies reported improvements in self-reported reading behavior (N=5/11), confidence in critical appraisal (N=7/7), critical appraisal test scores (N=5/7) and ability to use findings (N=5/7). No studies reported on patient outcomes [34]. |
| Communities of Practice (CoPs) | Pull Efforts | Li [37] – 3  LaRocca [15] – 7 | The systematic review by Li and colleagues found no studies that assessed the effectiveness of CoPs for healthcare practitioners [37]. The more recent systematic review by LaRocca and colleagues included one RCT that measured the impact of CoPs but there was no significant change in knowledge or practice of healthcare practitioners [15]. |
| Mentoring, Opinion leaders, Change agency | Pull Efforts | Abdullah [22] – 9  Flodgren [28] – 10  McCormack [39] – 2 | Only one study, with low risk of bias, compared a multifaceted intervention with mentoring to the same kind of intervention without mentoring (i.e. educational meetings combined with educational materials, and audit and feedback). This study showed mixed effects for practitioners’ behavior, with one outcome improving and others showing no difference [22]. Opinion leaders alone or in combination with other interventions may successfully promote evidence-based practice, but effectiveness varies both within and between studies [28]. Overall, across all 18 studies the median adjusted risk difference was +0.12 representing a 12% absolute increase in compliance in the intervention group [28]. A low quality systematic review suggested that change agents who are adequately supported and resourced (context) and who model the roles and practices they espouse (mechanism), have greater potential to achieve evidence-informed healthcare (outcome) [39]. |
| Toolkits, which include multiple resources (KT tools and strategies) for educating and/or facilitating behavior change.  Toolkits embedded in a multi-strategy intervention. | Climate, Push Efforts, Pull Efforts | Yamada [47] – 6 | The types of resources embedded within toolkits varied but included predominantly educational materials. Only four of five multi-strategy intervention studies demonstrated partial to mostly effective results in terms of clinical and/or implementation outcomes. Of the three single KT intervention studies, two were mostly effective at changing clinical outcomes. No studies evaluated the relative effectiveness of each KT strategy (e.g. use of audit and feedback); therefore, it was not possible to determine which components contributed to the change in outcomes [47]. |

CIRT – clinical information retrieval technology; CoPs – Communities of Practice; EBM – evidence-based medicine; EBP - evidence-based practice; KT – knowledge translation; PEMs – printed educational materials; RCT – randomized controlled trial.

* First author and reference

## Table 4 - Strategies tested in the policy environment only, quality scores, and main findings of included systematic reviews

| Strategy | Domain | Systematic review/s* and AMSTAR score | Findings |
| --- | --- | --- | --- |
| Interaction between users and producers of research | Linkage & Exchange | Moore [12] – 2 | No study provided evidence that interaction between researchers and policy makers has an impact on the use of research [12]. One matched case-control study found that units with interaction with researchers had a greater understanding of the report’s analyses and attached greater value to the report. Interaction was not associated with greater levels of utilization in terms of application [12]. |
| Access to systematic reviews | Facilitating pull efforts | Bunn [24] – 6  LaRocca [15] – 7  Moore [12] – 2  Murthy [42] – 9  Perrier [43] – 8  Wallace [45] – 8 | One RCT by Dobbins and colleagues [48] investigated three separate interventions: i) Access to systematic reviews and summaries (‘website’); ii) website plus tailored and targeted messages; and iii) website plus tailored and targeted messages plus knowledge brokers. It found that  Having access to a registry of synthesized and translated research evidence (website) had no impact on evidence-informed decision making (EIDM) (p<0.45);  Tailored and targeted messaging was significantly more effective in promoting EIDM than other strategies (p<0.009);  The KB was not effective overall but was more effective in those organizations that placed less value on research evidence and was less effective in those organizations that already recognized the importance of evidence-based decision making. The authors concluded that the KB intervention may not have contained all the necessary components to produce a positive effect. Also, for the group that worked with a knowledge broker, 30% of participants had limited or no engagement with the knowledge broker, thus the authors recommend caution with the generalizability of these results [48].  Simple knowledge translation and exchange strategies may be as effective as complex ones (but need to be active rather than passive);  Note that this same RCT was reported in all six systematic reviews. |
| Tailored and targeted messages | Push Efforts |  |  |
| Knowledge brokering (KB) | Linkage & Exchange |  |  |
| Disseminating evidence | Push Efforts | LaRocca [15] – 7 | Dissemination of information to school personnel, community providers, and policy makers via a printed pamphlet, CD-ROM or internet led to a significant improvement in knowledge but no change in practice in one RCT [15]. |
| Disseminating systematic reviews | Push Efforts | Perrier [43] – 8 | One study where five systematic reviews were mailed to public health officials and followed up with surveys at three months and two years reported that from 23% to 63% of respondents declared that they had used SRs in policymaking decisions [43]. |
| Training in the appraisal of research and its use | Pull Efforts | Moore [12] – 2 | One study that used self-assessment and no control group found that training of clinician managers, program managers and executives in the appraisal of research and its use appears to increase participants’ skills in critical appraisal and possibly their perceptions about the value of research, but not their use of research [12]. |
| Packaging of systematic reviews as summaries, overviews or policy briefs | Push Efforts, Facilitating Pull Efforts | Chambers [25] – 4 | Seven evaluation studies were included. No studies evaluated policy briefs. The quality of evidence is very low. The majority of studies reported on the perceived usefulness of the service, although there were some examples of review-based resources being used to assist actual decision making. However, the extent to which these resources are used and are found useful by policymakers is unclear [25]. |

EIDM – evidence-informed decision making; KB – knowledge brokering; RCT – randomized controlled trial.

* First author and reference

## Table 5 - Strategies tested in the policy and practice environment, quality scores, and main findings of included systematic reviews

| **Strategy** | **Domain** | **Systematic review/s* and AMSTAR score** | **Findings** |
| --- | --- | --- | --- |
| External inspection of compliance with standards | Climate | Flodgren [29] – 9 | Only two studies were identified for inclusion in the review, which highlights the paucity of high-quality controlled evaluations of the effectiveness of external inspection systems. No firm conclusions could therefore be drawn about the effectiveness of external inspection on compliance with standards [29]. |
| Mass mailing a printed bulletin which summarizes systematic review evidence | Push Efforts | Murthy [42] – 9 | Mass mailing a printed bulletin to NHS clinicians and decision-makers which summarizes systematic review evidence may improve evidence-based practice when there is a single clear message, if the change is relatively simple to accomplish, and there is a growing awareness by users of the evidence that a change in practice is required (3 interrupted time series studies) [42]. |
| Nurse manager education and/or support; change in policy, plans, structures; establishment of networks; leadership | Climate, Push Efforts, Pull Efforts | Gifford [31] – 4 | Four of the eight quantitative studies included evaluations of the effect of an intervention that included managers to promote nurses’ use of research evidence. Three studies showed positive results on nurse practice (2 before and after studies, 1 case study with a survey); however, lack of a control group makes it impossible to draw firm conclusions about the effects of these interventions. The RCT found no effect on selected patient outcomes. It would appear that leadership development at multiple levels is important to address the complexity of factors involved in nurses’ use of research, and should be a focus for future research [31]. |
| Multi-faceted interventions | Push Efforts, Facilitating Pull Efforts, Pull Efforts | LaRocca [15] – 7  Murthy [42] – 9  Perrier [44] – 9 | The multifaceted intervention that included education, a question and answer service and free access to databases lead to a significant improvement in knowledge but no change in practice [15]. Simple or single KT strategies were shown in some circumstances to be as effective as complex, multifaceted ones when changing practice, including tailored and targeted messaging [15]. There is insufficient evidence to support the other multifaceted interventions, including: multifaceted plus access to the WHO Reproductive Health Library; and Analgesic league table (based on systematic reviews) plus audit and feedback plus education [42, 44]. |

KT – knowledge translation; NHS – National Health Service; RCT – randomized controlled trial

* First author and reference

## Table 6. Summary of strategies for which there is some evidence of effectiveness on some outcomes in the practice and/or policy environment

| **Strategy** | **Outcomes improved** |
| --- | --- |
| **Practice** |  |
| Access to clinical information retrieval technology (e.g. Medline) [30, 38]; | Skills |
| Clinical librarian services [23]; | Clinical practice |
| Education in evidence-based practice (EBP) [27, 36]; | Knowledge  Skills  Attitudes  Behaviour towards EBP |
| Education in critical appraisal (low intensity) – two RCTs [35]; | Knowledge |
| Educational visits (outreach) plus donation of Cochrane Library and other materials – evidence from one RCT [44, 45]; | One clinical practice* |
| Dissemination of printed educational materials (PEM) [32]; | Clinical practice |
| Disseminating short summaries of systematic reviews – one RCT [45]; | Awareness |
| Multi-component strategies for dissemination of PEMs compared with single dissemination strategies [40]; | Clinical practice |
| Certain ways of communicating directness and net benefit in PEMs [40]; | Reduced uncertainty |
| Local opinion leaders alone or in combination with other interventions [28]; | Compliance with EBP |
| Some toolkits alone or embedded in a multi-strategy intervention [47]. | Clinical outcomes and/or  Implementation outcomes |
| **Policy** |  |
| Tailored and targeted messaging – evidence from one RCT [12, 15, 24, 42, 43, 45]; | Evidence-informed decision-making |
| Disseminating evidence – one RCT [15]; | Knowledge |
| Disseminating systematic reviews – one observational study [43] | Use of SRs in policy decision-making |
| Training in the appraisal of research and its use – one observational study [12]. | Skills |
| **Policy and practice** |  |
| Mass mailing a printed bulletin which summarizes systematic review evidence (when there is a single clear message, the change is relatively simple to accomplish, and there is a growing awareness by users of the evidence that a change in practice is required) – 3 interrupted time series studies [42] | EBP |
| A multifaceted intervention that included education, a question and answer service and free access to databases – one RCT [15] | Knowledge |
| Simple or single knowledge translation strategies were shown in some circumstances to be as effective as complex, multifaceted ones, including tailored and targeted messaging [15] | Practice |

EBP – evidence-based practice; PEM – printed educational materials; RCT – randomized controlled trial; SR – systematic review.

*Four clinical practices were measured in the study but only one of these improved significantly.

1. A range of terms are used in the literature, including framework, model, conceptual framework or model, theoretical framework or model and these terms are often used interchangeably without clear definition. [↑](#endnote-ref-1)
