# Supplementary material for: Designing a rapid response program to support evidence-informed decision-making in the Americas region: using the best available evidence and case studies
Source: Implement Sci. 2016 Aug 18;11:117. doi: 10.1186/s13012-016-0472-9 (PMC4990866; doi:10.1186/s13012-016-0472-9)
Supplement: Supplementary file 2 — Appendices to the rapid overview of strategies to facilitate evidence-informed decision-making. (DOCX 187 kb) [file 13012_2016_472_MOESM2_ESM.docx]

**Additional file 2 – Appendices to the rapid overview of strategies to facilitate evidence-informed decision making**

**Table of contents**

[Changes to the protocol 2](#_Toc449433119)

[Search terms 3](#_Toc449433120)

[Characteristics of excluded studies and reference details 6](#_Toc449433121)

[Table 1. Systematic reviews excluded from data extraction (n=14) 6](#_Toc449433122)

[References to systematic reviews that did not proceed to data extraction (n=14) 6](#_Toc449433123)

[Table 2. Excluded studies (n=78) 8](#_Toc449433124)

[References: Excluded studies (n=78) 10](#_Toc449433125)

[References to systematic reviews of barriers and facilitators excluded from data extraction (n=27) 16](#_Toc449433126)

[Characteristics of included studies 18](#_Toc449433127)

[Table 3a. Practice only (n=15) 18](#_Toc449433128)

[Table 3b. Policy & Practice (n=7) 24](#_Toc449433129)

[Table 3c. Policy only (n=5) 28](#_Toc449433130)

[Quality assessment of included systematic reviews 31](#_Toc449433131)

[Table 4a. Assessment of the included systematic reviews against the AMSTAR criteria – Practice only 31](#_Toc449433132)

[Table 4b. Assessment of the included systematic reviews against the AMSTAR criteria – Practice only 32](#_Toc449433133)

[Table 5. Assessment of the included systematic reviews against the AMSTAR criteria – Policy & Practice 33](#_Toc449433134)

[Table 6. Assessment of the included systematic reviews against the AMSTAR criteria – Policy only 34](#_Toc449433135)

[Shortcuts taken in this review to make it rapid and AMSTAR quality assessment 35](#_Toc449433136)

[Shortcuts taken 35](#_Toc449433137)

[Table 7. Assessment of this rapid review against the AMSTAR criteria 35](#_Toc449433138)

# Changes to the protocol

### Selection criteria:

- Clarifying that for ‘types of participants’ they needed to be within the field of health policy and practice.
- Types of articles/interventions: articles that evaluated barriers and/or facilitators to the uptake of research evidence were also sought, though this was not clearly specified in the published protocol.
- Outcomes: cost-effectiveness was not in the published protocol but was added during the study selection process as it was considered to be important for the overview.
- The search years were not limited to December 2014 and articles published in 2015 were included.

### Search:

- Medline (Ovid) was used instead of PubMed as PubMed did not allow the use of truncation characters in phrases.
- During the search process (but before study selection) it was decided to delete the terms: “decision-making” and “decision making” from the search strategy for MEDLINE and EMBASE as they were making the search far too sensitive and unmanageable.
- Searches of EconLit and NHSEED were not restricted to systematic reviews.
- A manual search of the Health Systems Evidence database was also undertaken in addition to the electronic search. This was done by using the advanced search option of ‘Topic’ and searching for recent high quality system reviews within each relevant category.

### Data extraction:

- Systematic reviews that were specific to a single health issue (e.g. pain management) or to a single profession (e.g. nursing) were excluded from data extraction in order to focus on the systematic reviews that were generalizable across health care issues and across professions. Further, where there were multiple systematic reviews addressing the same intervention or question, data were only extracted from the most recent good quality review(s). This was done to reduce the risk of double-counting of the same primary studies.
- Though part of the original protocol, we did not extract data on critical success factors, limitations and research gaps relevant to each included systematic review or primary study. This was due to time and resource limitations.

# Search terms

| **Database and search string** |
| --- |
| **CINAHL (EBSCOHost)**  S1 TI (“Information dissemination” OR “diffusion of innovation” OR “knowledge generation” OR “knowledge translation” OR “knowledge transfer” OR “knowledge uptake” OR “knowledge exchange” OR “knowledge broker*” OR “knowledge mobilization” OR “knowledge mobilisation” OR “research uptake” OR “research use” OR “use of research” OR “evidence-informed” OR “decision-making” OR “research utilization” OR “research utilisation” OR “technology transfer” OR “knowledge-to-action” OR “implementation science”)  S2 AB (“Information dissemination” OR “diffusion of innovation” OR “knowledge generation” OR “knowledge translation” OR “knowledge transfer” OR “knowledge uptake” OR “knowledge exchange” OR “knowledge broker*” OR “knowledge mobilization” OR “knowledge mobilisation” OR “research uptake” OR “research use” OR “use of research” OR “evidence-informed” OR “decision-making” OR “research utilization” OR “research utilisation” OR “technology transfer” OR “knowledge-to-action” OR “implementation science”)  S3 S1 OR S2  S4 TI ( “systematic review” OR "meta-analysis" OR MEDLINE ) OR AB ( “systematic review” OR "meta-analysis" OR MEDLINE )  S5 S3 AND S4  **Limiters** - Published Date: 20040101-20151231 |
| **Cochrane Library**  **Cochrane Database of Systematic Reviews, DARE, HTA database, NHSEED**  #1 "Information dissemination":ti,ab,kw or "diffusion of innovation":ti,ab,kw or "knowledge generation":ti,ab,kw or "knowledge translation":ti,ab,kw or "knowledge transfer":ti,ab,kw (Word variations have been searched)  #2 "knowledge uptake":ti,ab,kw or "knowledge exchange":ti,ab,kw or knowledge next broker*:ti,ab,kw or knowledge next mobili?ation:ti,ab,kw or "research uptake":ti,ab,kw  #3 research near/2 use:ti,ab,kw or evidence-informed:ti,ab,kw or decision-making:ti,ab,kw or research next utili?ation:ti,ab,kw or technology next transfer:ti,ab,kw  #4 knowledge-to-action:ti,ab,kw or implementation next science:ti,ab,kw (Word variations have been searched)  #5 #1 OR #2 OR #3 OR #4  **Cochrane Database of Methods Studies** (above plus:)  #6 systematic next review:ti,ab,kw or meta-analysis:ti,ab,kw or MEDLINE:ti,ab,kw  #7 #5 and #6 |
| **EconLit (EBSCOHost)**  S1 TI (“Information dissemination” OR “diffusion of innovation” OR “knowledge generation” OR “knowledge translation” OR “knowledge transfer” OR “knowledge uptake” OR “knowledge exchange” OR “knowledge broker*” OR “knowledge mobilization” OR “knowledge mobilisation” OR “research uptake” OR “research use” OR “use of research” OR “evidence-informed” OR “decision-making” OR “research utilization” OR “research utilisation” OR “technology transfer” OR “knowledge-to-action” OR “implementation science”)  S2 AB (“Information dissemination” OR “diffusion of innovation” OR “knowledge generation” OR “knowledge translation” OR “knowledge transfer” OR “knowledge uptake” OR “knowledge exchange” OR “knowledge broker*” OR “knowledge mobilization” OR “knowledge mobilisation” OR “research uptake” OR “research use” OR “use of research” OR “evidence-informed” OR “decision-making” OR “research utilization” OR “research utilisation” OR “technology transfer” OR “knowledge-to-action” OR “implementation science”)  S3 S1 OR S2  S4 SU cost-benefit analysis OR SU cost-effectiveness OR SU cost-utility  S5 S3 AND S4  S6 S3 AND S4  **Limiters** - Published Date: 20040101-20151231 |
| **EMBASE (Ovid)**   1. (Information dissemination or diffusion of innovation or knowledge generation or knowledge translation or knowledge transfer or knowledge uptake or knowledge exchange or knowledge broker$ or knowledge mobilization or knowledge mobilisation).mp. [mp=title, abstract, original title, name of substance word, subject heading word, keyword heading word, protocol supplementary concept word, rare disease supplementary concept word, unique identifier] 2. (research adj3 "use").mp. 3. (research uptake or evidence informed or evidence-informed or research utilization or research utilisation or technology transfer or implementation science).mp. 4. (knowledge adj2 action).mp. 5. 1 or 2 or 3 or 4 6. (systematic review or meta-analysis or MEDLINE).mp. 7. 5 and 6 8. limit 7 to yr="2004 -Current" |
| **Health Systems Evidence**^[[1]](#footnote-1)^  (Information dissemination OR diffusion of innovation OR knowledge generation OR knowledge translation OR knowledge transfer OR knowledge uptake OR knowledge exchange OR knowledge broker OR knowledge mobilization OR knowledge mobilisation OR research uptake OR research use OR use of research OR evidence informed OR evidence-informed OR decision making OR decision-making OR research utilization OR research utilisation OR technology transfer OR knowledge-to-action OR knowledge to action OR implementation science) In title or abstract.  Restrict to systematic reviews. |
| **LILACS (BVSalud)**  (tw:(“Information dissemination” )) OR (tw:(“diffusion of innovation” )) OR (tw:(“knowledge generation”)) OR (tw:(“knowledge translation”)) OR (tw:(“knowledge transfer” )) OR (tw:(“knowledge uptake”)) OR (tw:(“knowledge exchange”)) OR (tw:(knowledge broker$)) OR (tw:(“knowledge mobilization”)) OR (tw:(“knowledge mobilisation” )) OR (tw:(“research uptake”)) OR (tw:(“research use”)) OR (tw:(“use of research”)) OR (tw:(“evidence informed”)) OR (tw:(“evidence-informed”)) OR (tw:(“decision making”)) OR (tw:(“decision-making”)) OR (tw:(“research utilization”)) OR (tw:(“research utilisation”)) OR (tw:(“technology transfer”)) OR (tw:(“knowledge-to-action”)) OR (tw:(“knowledge to action”)) OR (tw:(“implementation science”)) |
| **MEDLINE (Ovid)**   1. (Information dissemination or diffusion of innovation or knowledge generation or knowledge translation or knowledge transfer or knowledge uptake or knowledge exchange or knowledge broker$ or knowledge mobilization or knowledge mobilisation).mp. [mp=title, abstract, original title, name of substance word, subject heading word, keyword heading word, protocol supplementary concept word, rare disease supplementary concept word, unique identifier] 2. (research adj3 "use").mp. 3. (research uptake or evidence informed or evidence-informed or research utilization or research utilisation or technology transfer or implementation science).mp. 4. (knowledge adj2 action).mp. 5. 1 or 2 or 3 or 4 6. (systematic review or meta-analysis or MEDLINE).mp. 7. 5 and 6 8. limit 7 to yr="2004 -Current" |
| **Website and search terms** |
| **Google / Google Scholar**  information dissemination AND systematic review  knowledge translation AND systematic review  research use AND systematic review  research uptake AND systematic review |

# Characteristics of excluded studies and reference details

## Table 1. Systematic reviews excluded from data extraction (n=14)

| **Reason for exclusion from data extraction** | **Systematic reviews** |
| --- | --- |
| Specific to a single health issue | (MacGregor et al. 2014, Ospina et al. 2013, Schleifer Taylor et al. 2014) |
| Specific to a single profession | (Flodgren et al. 2012, Menon et al. 2009, Scott et al. 2012, Thompson et al. 2007, Wuchner 2014). |
| Older systematic review addressing the same intervention/s or question as recent good quality reviews | (Armstrong 2011, Armstrong et al. 2013, Coomarasamy and Khan 2004, Flores-Mateo and Argimon 2007, Wagner and Byrd 2004, Weightman et al. 2005). |

## References to systematic reviews that did not proceed to data extraction (n=14)

Armstrong R. 2011. *Evidence-informed public health decision-making in local government, PhD Thesis.* Melbourne School of Public Health, The University of Melbourne.

Armstrong R, Waters E, Dobbins M, Anderson L, Moore L, Petticrew M, et al. 2013. Knowledge translation strategies to improve the use of evidence in public health decision making in local government: intervention design and implementation plan. *Implementation Science* 8; 121.

Coomarasamy A and Khan KS 2004. What is the evidence that postgraduate teaching in evidence based medicine changes anything? A systematic review. *BMJ* 329; 1017.

Flodgren G, Rojas-Reyes MX, Cole N and Foxcroft DR 2012. Effectiveness of organisational infrastructures to promote evidence-based nursing practice. *Cochrane Database of Systematic Reviews* Issue 2; Art. No. CD002212.

Flores-Mateo G and Argimon JM 2007. Evidence based practice in postgraduate healthcare education: a systematic review. *BMC Health Services Research* 7; 119.

MacGregor JC, Wathen N, Kothari A, Hundal PK and Naimi A 2014. Strategies to promote uptake and use of intimate partner violence and child maltreatment knowledge: an integrative review. *BMC Public Health* 14; 862.

Menon A, Korner-Bitensky N, Kastner M, McKibbon KA and Straus S 2009. Strategies for rehabilitation professionals to move evidence-based knowledge into practice: a systematic review. *Journal of Rehabilitation Medicine* 41; 1024-1032.

Ospina MB, Taenzer P, Rashiq S, MacDermid JC, Carr E, Chojecki D, et al. 2013. A systematic review of the effectiveness of knowledge translation interventions for chronic noncancer pain management. *Pain Research & Management* 18; e129-141.

Schleifer Taylor J, Verrier MC and Landry MD 2014. What do we know about knowledge brokers in paediatric rehabilitation? A systematic search and narrative summary. *Physiotherapy Canada* 66; 143-152.

Scott SD, Albrecht L, O'Leary K, Ball GD, Hartling L, Hofmeyer A, et al. 2012. Systematic review of knowledge translation strategies in the allied health professions. *Implementation Science* 7; 70.

Thompson DS, Estabrooks CA, Scott-Findlay S, Moore K and Wallin L 2007. Interventions aimed at increasing research use in nursing: a systematic review. *Implementation Science* 2; 15.

Wagner KC and Byrd GD 2004. Evaluating the effectiveness of clinical medical librarian programs: a systematic review of the literature. *Journal of the Medical Library Association* 92; 14-33.

Weightman AL, Williamson J, Library, Knowledge Development Network Q and Statistics G 2005. The value and impact of information provided through library services for patient care: a systematic review. *Health Information & Libraries Journal* 22; 4-25.

Wuchner SS 2014. Integrative review of implementation strategies for translation of research-based evidence by nurses. *Clinical Nurse Specialist* 28; 214-223.

## Table 2. Excluded studies (n=78)

| **Study** | **Reason for Exclusion** |
| --- | --- |
| Ahmadi et al. 2015 | Participants - not yet practicing health professionals; Intervention |
| Arditi et al. 2012 | Intervention - not a KT strategy (reminder did not have to be based on evidence). |
| [Armstrong et al. 2011](#_ENREF_1) | Study Type - protocol for a relevant systematic review. |
| [Barbui et al. 2014](#_ENREF_2) | Intervention - not a KT strategy. |
| [Barosi 2006](#_ENREF_3) | Study Type - narrative review; Outcomes - not measured. |
| [Barwick et al. 2012](#_ENREF_4) | Intervention - not a KT strategy. |
| [Boaz et al. 2011](#_ENREF_5) | Study Type - overview of systematic reviews. |
| [Boersma et al. 2015](#_ENREF_6) | Intervention - not a KT strategy; Outcomes. |
| [Bostrom et al. 2012](#_ENREF_7) | Outcomes not reported. |
| [Braithwaite et al. 2014](#_ENREF_8) | Intervention - not a KT strategy; Outcomes. |
| [Chaillet et al. 2006](#_ENREF_9) | Intervention - not a KT strategy; Outcomes. |
| [Child et al. 2012](#_ENREF_10) | Intervention - not KT strategy; Outcomes - not focused on KT. |
| [Christie et al. 2012](#_ENREF_11) | Study Type - not a systematic review (no results section). |
| [Clay-Williams et al. 2014](#_ENREF_12) | Intervention - not a KT strategy; Outcomes. |
| [Contandriopoulos 2012](#_ENREF_13) | Study Type - narrative review of the 2010 systematic review by the same author; Outcomes - not measured. |
| [Contandriopoulos et al. 2010](#_ENREF_14) | Outcomes - not measured. |
| [Crilly et al. 2013](#_ENREF_15) | Outcomes - not measured. |
| [Cummings et al. 2011](#_ENREF_16) | Intervention - not a KT strategy; Outcomes. |
| [Cummings et al. 2008](#_ENREF_17) | Duplicate of above study (Cummings et al 2008) |
| [Desomer et al. 2013](#_ENREF_18) | Study Type - systematic review of systematic reviews, not primary studies. |
| [Divall et al. 2013](#_ENREF_19) | Intervention - not a KT strategy. |
| Dulko 2007 | Study type - not a SR - one reviewer, no detail on selection and data extraction; Intervention - not specifically focussed on research use. |
| Elueze 2015 | Study type - only one reviewer / 'systematised' / mostly narrative. |
| Fiander et al. 2015 | Intervention - not specifically focussed on research use. |
| [Field et al. 2014](#_ENREF_20) | Effectiveness not measured; Outcomes not measured. |
| [Fillion and Rochette 2010](#_ENREF_21) | Outcomes |
| [Fitzgerald et al. 2014](#_ENREF_22) | Study Type - systematic review of systematic reviews |
| [Flodgren et al. 2011](#_ENREF_23) | Study Type - systematic review of systematic reviews;  Intervention - not a KT strategy; Outcomes. |
| [Francke et al. 2008](#_ENREF_25) | Study Type - systematic review of systematic reviews; No intervention; |
| [Fung et al. 2008](#_ENREF_26) | Intervention - not a KT strategy; Outcomes. |
| [Gagliardi et al. 2014](#_ENREF_27) | Intervention - not a KT strategy; Outcomes. |
| [Gagnon et al. 2014](#_ENREF_28) | Intervention - not a KT strategy, guideline implementation, not necessarily based on evidence. |
| Gerhardus & Dintsious 2005 | Language - German |
| [Goldner et al. 2011](#_ENREF_29) | Study Type - scoping review; Outcomes not reported. |
| [Goldner et al. 2014](#_ENREF_30) | Study Type - systematic review of reviews. |
| [Grimshaw et al. 2004a](#_ENREF_31) | Study Type - narrative review; Outcomes not measured. |
| [Grimshaw et al. 2004b](#_ENREF_32) | Intervention - not a KT strategy. |
| [Haines et al. 2004](#_ENREF_33) | Study Type - narrative review. |
| [Hampton et al. 2014](#_ENREF_34) | Intervention - not a KT strategy, guideline implementation, not necessarily based on evidence. |
| [Haynes et al. 2010](#_ENREF_35) | Study Type - protocol for a systematic review; Intervention - not a KT strategy. |
| [Hemens et al. 2011](#_ENREF_36) | Intervention - not a KT strategy. |
| Holt et al. 2012 | Intervention - not specifically focused on research use. |
| Ivers et al. 2012 | Intervention - not specifically focused on research use. |
| [John et al. 2014](#_ENREF_37) | Duplicate of Wallace et al 2014. |
| [Ketelaar et al. 2011](#_ENREF_38) | Intervention - not a KT strategy; Outcomes. |
| [Lamb et al. 2011](#_ENREF_39) | Intervention - not a KT strategy; Outcomes. |
| [Lemire et al. 2013](#_ENREF_40) | Intervention - not a KT strategy; Outcomes - not measured. |
| [Mairs et al. 2013](#_ENREF_41) | Outcomes |
| [Menon et al. 2010a](#_ENREF_42) | Abstract only. Full version available (Menon et al 2009). |
| [Menon et al. 2010b](#_ENREF_43) | Abstract only. Full version available (Menon et al 2009). |
| [Milner et al. 2006](#_ENREF_44) | Intervention - did not measure the effectiveness of KT strategies; Outcomes - not measured |
| [Moat et al. 2013](#_ENREF_45) | Outcomes - views of policy-makers regarding policy briefs. |
| [Morgan 2010](#_ENREF_46) | Study Type - systematic review of reviews (not necessarily systematic reviews); Intervention - not a KT strategy; Outcomes - not measured. |
| [Novins et al. 2013](#_ENREF_47) | Intervention - not a KT strategy. |
| [Olade 2004](#_ENREF_48) | Study Type - narrative review, not systematic review. |
| [Orton et al. 2010a](#_ENREF_49) | Abstract only. Full version available (Orton et al 2011). |
| [Orton et al. 2010b](#_ENREF_50) | Abstract only. Full version available (Orton et al 2011). |
| Parmelli et al. 2011 | Intervention - did not have to be supported by research evidence. |
| Pentland et al. 2011 | Study Type - this is a systematic review of overviews, systematic reviews, narrative reviews and some primary studies with weak study designs (qualitative). |
| Quinn et al. 2014 | Outcomes - not focused on research uptake. |
| [Rajic et al. 2013](#_ENREF_51) | Outcomes - not measured. |
| [Riley et al. 2012](#_ENREF_52) | Study Type - not a systematic review. |
| Rankin et al. 2008 | Intervention - not specifically focused on research use. |
| Ranmuthugala et al. 2011a | Study type - protocol for a systematic review. |
| Ranmuthugala et al. 2011b | Intervention - not specifically focused on research use. |
| Rohrbasser et al. 2013 | Study type - protocol for a systematic review. |
| Rotter et al. 2010 | Intervention - clinical pathway did not have to be based on evidence. |
| [Scott et al. 2011](#_ENREF_53) | Study Type - protocol for an included systematic review (Scott et al 2012). |
| [Scott et al. 2013](#_ENREF_54) | Study Type - protocol for a systematic review. |
| [Siddiqi et al. 2005](#_ENREF_55) | Intervention - not a KT strategy. |
| [Sinuff et al. 2013](#_ENREF_56) | Intervention - not a KT strategy. |
| [Smolders et al. 2008](#_ENREF_57) | Intervention - not a KT strategy. |
| [Smylie 2014](#_ENREF_58) | Study Type - not a systematic review; Outcomes. |
| [Stirman et al. 2012](#_ENREF_59) | Intervention - not a KT strategy; Outcomes. |
| [Thompson and Stapley 2011](#_ENREF_60) | Intervention - not a KT strategy; Outcomes. |
| [Tricco et al. 2013](#_ENREF_61) | Study Type - protocol for a scoping systematic review; Outcomes will not be measured. |
| Wong et al. 2013 | Participants - not yet practicing health professionals; Intervention |
| Young et al. 2014 | Study type - overview of SRs. Used as a source of SRs. |
| [Zwarenstein and Reeves 2006](#_ENREF_62) | Intervention - not a KT strategy; Outcomes. |

## References: Excluded studies (n=78)

Ahmadi SF, Baradaran HR and Ahmadi E 2015. Effectiveness of teaching evidence-based medicine to undergraduate medical students: a BEME systematic review. *Medical Teacher* 37; 21-30.

Arditi C, Rege-Walther M, Wyatt JC, Durieux P and Burnand B 2012. Computer-generated reminders delivered on paper to healthcare professionals; effects on professional practice and health care outcomes. *Cochrane Database of Systematic Reviews* 12; CD001175.

Armstrong R, Waters E, Dobbins M, Lavis John N, Petticrew M and Christensen R. 2011. Knowledge translation strategies for facilitating evidence-informed public health decision making among managers and policy-makers [Protocol]. *Cochrane Database of Systematic Reviews* [Online]. Available: http://onlinelibrary.wiley.com/doi/10.1002/14651858.CD009181/abstract.

Barbui C, Girlanda F, Ay E, Cipriani A, Becker T and Koesters M. 2014. Implementation of treatment guidelines for specialist mental health care. *Cochrane Database of Systematic Reviews* [Online]. Available: http://onlinelibrary.wiley.com/doi/10.1002/14651858.CD009780.pub2/abstract

Barosi G 2006. Strategies for dissemination and implementation of guidelines. *Neurological Sciences* 27 Suppl 3; S231-4.

Barwick MA, Schachter HM, Bennett LM, McGowan J, Ly M, Wilson A, et al. 2012. Knowledge translation efforts in child and youth mental health: a systematic review. *Journal of Evidence-Based Social Work* 9; 369-95.

Boaz A, Baeza J, Fraser A and European Implementation Score Collaborative G 2011. Effective implementation of research into practice: an overview of systematic reviews of the health literature. *BMC Research Notes* 4; 212.

Boersma P, van Weert JC, Lakerveld J and Droes RM 2015. The art of successful implementation of psychosocial interventions in residential dementia care: a systematic review of the literature based on the RE-AIM framework. *International Psychogeriatrics* 27; 19-35.

Bostrom AM, Slaughter SE, Chojecki D and Estabrooks CA 2012. What do we know about knowledge translation in the care of older adults? A scoping review. *Journal of the American Medical Directors Association* 13; 210-9.

Braithwaite J, Marks D and Taylor N 2014. Harnessing implementation science to improve care quality and patient safety: a systematic review of targeted literature. *International Journal for Quality in Health Care* 26; 321-9.

Chaillet N, Dube E, Dugas M, Audibert F, Tourigny C, Fraser WD, et al. 2006. Evidence-based strategies for implementing guidelines in obstetrics: a systematic review (Structured abstract). *Obstetrics and Gynecology* [Online], 108. Available: http://onlinelibrary.wiley.com/o/cochrane/cldare/articles/DARE-12006007867/frame.html

Child S, Goodwin V, Garside R, Jones-Hughes T, Boddy K and Stein K 2012. Factors influencing the implementation of fall prevention programmes: A systematic review and synthesis of qualitative studies. *Implementation Science* 7; 91.

Christie J, Hamill C and Power J 2012. How can we maximize nursing students' learning about research evidence and utilization in undergraduate, preregistration programmes? A discussion paper. *Journal of Advanced Nursing* 68; 2789-801.

Clay-Williams R, Nosrati H, Cunningham FC, Hillman K and Braithwaite J 2014. Do large-scale hospital- and system-wide interventions improve patient outcomes: a systematic review. *BMC Health Services Research* 14; 369.

Contandriopoulos D 2012. Some Thoughts on the Field of KTE. *Healthcare Policy = Politiques de sante* 7; 29-37.

Contandriopoulos D, Lemire M, Denis JL and Tremblay E 2010. Knowledge exchange processes in organizations and policy arenas: a narrative systematic review of the literature. *Milbank Quarterly* 88; 444-83.

Crilly T, Jashapara A, Trenholm S, Peckham A, Currie G and Ferlie E 2013. *Knowledge mobilisation in healthcare organisations: Synthesising evidence and theory using perspectives of organisational form, resource based view of the firm and critical theory.* National Institute for Health Research.

Cummings GG, Armijo Olivo S, Biondo PD, Stiles CR, Yurtseven O, Fainsinger RL, et al. 2011. Effectiveness of knowledge translation interventions to improve cancer pain management. *Journal of Pain and Symptom Management* 41; 915-939.

Cummings GG, Hagen NA, Fainsinger R, Olivo SA, Stiles C and Biondo P 2008. Effectiveness of knowledge translation interventions to improve cancer pain management (Protocol). *Cochrane Database of Systematic Reviews* (4).

Desomer A, Dilles T, Steckel S, Duchesnes C, Vanmeerbeek M, Peremans L, et al. 2013. Dissemination and implementation of clinical practice guidelines in Belgium (Structured abstract). *Health Technology Assessment Database* [Online]. Available: http://onlinelibrary.wiley.com/o/cochrane/clhta/articles/HTA-32014001133/frame.html.

Divall P, Camosso-Stefinovic J and Baker R 2013. The use of personal digital assistants in clinical decision making by health care professionals: a systematic review. *Health Informatics Journal* 19; 16-28.

Dulko D 2007. Audit and feedback as a clinical practice guideline implementation strategy: a model for acute care nurse practitioners. *Worldviews on Evidence-Based Nursing* 4; 200-9.

Elueze IN 2015. Evaluating the effectiveness of knowledge brokering in health research: a systematised review with some bibliometric information. *Health Information & Libraries Journal*.

Fiander M, McGowan J, Grad R, Pluye P, Hannes K, Labrecque M, et al. 2015. Interventions to increase the use of electronic health information by healthcare practitioners to improve clinical practice and patient outcomes. *Cochrane Database of Systematic Reviews* 3; CD004749.

Field B, Booth A, Ilott I and Gerrish K 2014. Using the Knowledge to Action Framework in practice: a citation analysis and systematic review. *Implementation Science* 9; 172.

Fillion B and Rochette A 2010. Moving beyond knowledge translation to evidence-based practice by clinicians in neurology/stroke programs: Interaction between clinic and research. *Stroke* 41 (7); e506.

Fitzgerald A, Lethaby A, Cikalo M, Glanville J and Wood H 2014. Review of systematic reviews exploring the implementation/uptake of guidelines. The University of York.

Flodgren G, Eccles MP, Shepperd S, Scott A, Parmelli E and Beyer FR 2011. An overview of reviews evaluating the effectiveness of financial incentives in changing healthcare professional behaviours and patient outcomes. *Cochrane Database of Systematic Reviews*.

Francke AL, Smit MC, de Veer AJ and Mistiaen P 2008. Factors influencing the implementation of clinical guidelines for health care professionals: a systematic meta-review. *BMC Medical Informatics and Decision Making* 8; 38.

Fung CH, Lim YW, Mattke S, Damberg C and Shekelle PG 2008. Systematic review: the evidence that publishing patient care performance data improves quality of care. *Annals of Internal Medicine* 148; 111-23.

Gagliardi AR, Webster F, Perrier L, Bell M and Straus S 2014. Exploring mentorship as a strategy to build capacity for knowledge translation research and practice: a scoping systematic review. *Implementation Science* 9; 122.

Gagnon MM, Hadjistavropoulos T, Hampton A and Stinson J 2014. A systematic review of knowledge translation in pediatric pain. *Pain Research and Management* 19 (3); e73.

Gerhardus A and Dintsios CM 2005. [The influence of Health Technology Assessment reports on health policy decision-making - a systematic review]. Der Einfluss von HTA-Berichten auf die gesundheitspolitische Entscheidungsfindung: eine systematische Übersichtsarbeit. *GMS Health Technology Assessment.*

Goldner EM, Jeffries V, Bilsker D, Jenkins E, Menear M and Petermann L 2011. Knowledge translation in mental health: A scoping review. *Healthcare Policy* 7; 83-121.

Goldner EM, Jenkins EK and Fischer B 2014. A narrative review of recent developments in knowledge translation and implications for mental health care providers. *Canadian Journal of Psychiatry - Revue Canadienne de Psychiatrie* 59; 160-9.

Grimshaw J, Eccles M and Tetroe J 2004. Implementing clinical guidelines: current evidence and future implications. *Journal of Continuing Education in the Health Professions* 24 Suppl 1; S31-7.

Grimshaw JM, Thomas RE, MacLennan G, Fraser C, Ramsay CR, Vale L, et al. 2004. Effectiveness and efficiency of guideline dissemination and implementation strategies. *Health Technology Assessment* 8; iii-iv, 1-72.

Haines A, Kuruvilla S and Borchert M 2004. Bridging the implementation gap between knowledge and action for health. *Bulletin of The World Health Organization* 82; 724-31; discussion 732.

Hampton ADJ, Gagnon MM and Hadjistavropoulos T 2014. Knowledge translation and chronic pain in older adults: A systematic review. *Pain Research and Management* 19 (3); e76-e77.

Haynes RB, Wilczynski NL and Computerized Clinical Decision Support System Systematic Review T 2010. Effects of computerized clinical decision support systems on practitioner performance and patient outcomes: methods of a decision-maker-researcher partnership systematic review. *Implementation Science* 5; 12.

Hemens BJ, Holbrook A, Tonkin M, Mackay JA, Weise-Kelly L, Navarro T, et al. 2011. Computerized clinical decision support systems for drug prescribing and management: A decision-maker-researcher partnership systematic review. *Implementation Science* 6; 89.

Holt TA, Thorogood M and Griffiths F 2012. Changing clinical practice through patient specific reminders available at the time of the clinical encounter: systematic review and meta-analysis. *Journal of General Internal Medicine* 27; 974-984.

Ivers N, Jamtvedt G, Flottorp S, Young JM, Odgaard-Jensen J, French SD, et al. 2012. Audit and feedback: effects on professional practice and healthcare outcomes. *Cochrane Database of Systematic Reviews* 6; CD000259.

John W, Clarke C and Byrne C 2014. EPA-0139 – Increasing the uptake of systematic reviews: a systematic review of intervention effectiveness and relevance [abstract]. *European Psychiatry* 29 Suppl 1; 1.

Ketelaar NA, Faber MJ, Flottorp S, Rygh LH, Deane KH and Eccles MP 2011. Public release of performance data in changing the behaviour of healthcare consumers, professionals or organisations. *Cochrane Database of Systematic Reviews*; CD004538.

Lamb BW, Brown KF, Nagpal K, Vincent C, Green JS and Sevdalis N. 2011. Quality of care management decisions by multidisciplinary cancer teams: a systematic review (Structured abstract). *Annals of Surgical Oncology* [Online], 18. Available: http://onlinelibrary.wiley.com/o/cochrane/cldare/articles/DARE-12011005092/frame.html

Lemire M, Payette-Demers O and Jefferson-Falardeau J 2013. Dissemination of performance information and continuous improvement: A narrative systematic review. *Journal of Health Organization and Management* 27; 449-478.

Mairs K, McNeil H, McLeod J, Prorok JC and Stolee P 2013. Online strategies to facilitate health-related knowledge transfer: a systematic search and review. *Health Information & Libraries Journal* 30; 261-77.

Menon A, Korner-Bitensky N, Kastner M, McKibbon AK and Straus SE 2010. Knowledge translation strategies for promoting best practices in stroke rehabilitation. *Canadian Journal of Neurological Sciences* 1); S104.

Menon A, Korner-Bitensky N, Kastner M, McKibbon AK and Straus SE 2010. Knowledge translation strategies for promoting best practices in stroke rehabilitation. *Stroke* 41 (7); e508.

Milner M, Estabrooks CA and Myrick F. 2006. Research utilization and clinical nurse educators: a systematic review. *Journal of Evaluation in Clinical Practice* [Online], 12. Available: http://onlinelibrary.wiley.com/o/cochrane/clcmr/articles/CMR-13640/frame.html

Moat KA, Lavis JN and Abelson J 2013. How contexts and issues influence the use of policy-relevant research syntheses: A critical interpretive synthesis. *Milbank Quarterly* 91; 604-648.

Morgan G 2010. Evidence-based health policy: A preliminary systematic review. *Health Education Journal* 69; 43-47.

Novins DK, Green AE, Legha RK and Aarons GA 2013. Dissemination and implementation of evidence-based practices for child and adolescent mental health: a systematic review. *Journal of the American Academy of Child and Adolescent Psychiatry* 52; 1009-1025.e18.

Olade RA 2004. Strategic collaborative model for evidence-based nursing practice. *Worldviews on Evidence-Based Nursing* 1; 60-8.

Orton L, Lloyd-Williams F, Taylor-Robinson D, O'Flaherty M and Capewell S 2010. 052 Systematic review: the use of research evidence by public health policy-makers. *Journal of Epidemiology and Community Health* 64; A21-A21.

Orton L, Lloyd-Williams F, Taylor-Robinson D, O'Flaherty M and Capewell S. 2010. Systematic review: the use of research evidence by public health policy-makers [abstract]. *Journal of Epidemiology and Community Health* [Online], 64. Available: http://onlinelibrary.wiley.com/o/cochrane/clcmr/articles/CMR-16213/frame.html.

Parmelli E, Flodgren G, Beyer F, Baillie N, Schaafsma ME and Eccles MP 2011. The effectiveness of strategies to change organisational culture to improve healthcare performance: a systematic review. *Implementation Science* 6; 33.

Pentland D, Forsyth K, Maciver D, Walsh M, Murray R, Irvine L, et al. 2011. Key characteristics of knowledge transfer and exchange in healthcare: integrative literature review. *Journal of Advanced Nursing* 67; 1408-1425.

Quinn E, Huckel-Schneider C, Campbell D, Seale H and Milat AJ 2014. How can knowledge exchange portals assist in knowledge management for evidence-informed decision making in public health? *BMC Public Health* 14; 443.

Rajic A, Young I and McEwen SA 2013. Improving the utilization of research knowledge in agri-food public health: A mixed-method review of knowledge translation and transfer. *Foodborne Pathogens and Disease* 10; 397-412.

Rankin JA, Grefsheim SF and Canto CC 2008. The emerging informationist specialty: a systematic review of the literature. *Journal of the Medical Library Association* 96; 194-206.

Ranmuthugala G, Cunningham FC, Plumb JJ, Long J, Georgiou A, Westbrook JI, et al. 2011. A realist evaluation of the role of communities of practice in changing healthcare practice. *Implementation Science* 6; 49.

Ranmuthugala G, Plumb JJ, Cunningham FC, Georgiou A, Westbrook JI and Braithwaite J 2011. How and why are communities of practice established in the healthcare sector? A systematic review of the literature. *BMC Health Services Research* 11; 273.

Riley B, Norman CD and Best A 2012. Knowledge integration in public health: A rapid review using systems thinking. *Evidence and Policy* 8; 417-431.

Rohrbasser A, Mickan S and Harris J 2013. Exploring why quality circles work in primary health care: a realist review protocol. *Systematic Reviews* 2; 110.

Rotter T, Kinsman L, James E, Machotta A, Gothe H, Willis J, et al. 2010. Clinical pathways: effects on professional practice, patient outcomes, length of stay and hospital costs. *Cochrane Database of Systematic Reviews*; CD006632.

Scott SD, Albrecht L, O'Leary K, Ball GD, Dryden DM, Hartling L, et al. 2011. A protocol for a systematic review of knowledge translation strategies in the allied health professions. *Implementation Science* 6; 58.

Scott SD, Brett-MacLean P, Archibald M and Hartling L 2013. Protocol for a systematic review of the use of narrative storytelling and visual-arts-based approaches as knowledge translation tools in healthcare. *Systematic Reviews* 2; 19.

Siddiqi K, Newell J and Robinson M 2005. Getting evidence into practice: What works in developing countries? *International Journal for Quality in Health Care* 17; 447-454.

Sinuff T, Muscedere J, Adhikari NKJ, Stelfox HT, Dodek P, Heyland DK, et al. 2013. Knowledge translation interventions for critically ill patients: A systematic review. *Critical Care Medicine* 41; 2627-2640.

Smolders M, Laurant M, Roberge P, Van Balkom A, Van Rijswijk E, Bower P, et al. 2008. Knowledge transfer and improvement of primary and ambulatory care for patients with anxiety. *Canadian Journal of Psychiatry* 53; 277-293.

Smylie L 2014. Closing the gap: Translating research evidence on social determinants into practice to reduce health disparities in sexually transmitted and blood borne infections. *Sexually Transmitted Diseases* 41; S62.

Stirman WS, Kimberly J, Cook N, Calloway A, Castro F and Charms M 2012. The sustainability of new programs and innovations: A review of the empirical literature and recommendations for future research. *Implementation Science* 7; 17.

Thompson C and Stapley S 2011. Do educational interventions improve nurses’ clinical decision making and judgement? A systematic review. *International Journal of Nursing Studies* 48; 881-893.

Tricco AC, Cogo E, Ashoor H, Perrier L, McKibbon KA, Grimshaw JM, et al. 2013. Sustainability of knowledge translation interventions in healthcare decision-making: protocol for a scoping review. *BMJ Open* 3.

Wong SC, McEvoy MP, Wiles LK and Lewis LK 2013. Magnitude of change in outcomes following entry-level evidence-based practice training: a systematic review. *International Journal of Medical Education* 4; 107-114.

Young T, Rohwer A, Volmink J and Clarke M 2014. What are the effects of teaching evidence-based health care (EBHC)? Overview of systematic reviews. *PloS One* 9; e86706.

Zwarenstein M and Reeves S 2006. Knowledge translation and interprofessional collaboration: Where the rubber of evidence-based care hits the road of teamwork. *Journal of Continuing Education in the Health Professions* 26; 46-54.

## References to systematic reviews of barriers and facilitators excluded from data extraction (n=27)

Bunn F and Sworn K 2011. Strategies to promote the impact of systematic reviews on healthcare policy: A systematic review of the literature. *Evidence & Policy* 7; 403-428.

Clar C, Campbell S, Davidson L and Graham W 2011. What are the effects of interventions to improve the uptake of evidence from health research into policy in low and middle-income countries? Final Report to DFID. Ref.No. (PO) 40032846. London, UK: Research for Development (R4D), Department for International Development.

Cochrane LJ, Olson CA, Murray S, Dupuis M, Tooman T and Hayes S 2007. Gaps between knowing and doing: understanding and assessing the barriers to optimal health care. *Journal of Continuing Education in the Health Professions* 27; 94-102.

Eddama O and Coast J 2008. A systematic review of the use of economic evaluation in local decision-making. *Health Policy* 86; 129-141.

Gifford W, Davies B, Edwards N, Griffin P and Lybanon V 2007. Managerial leadership for nurses' use of research evidence: an integrative review of the literature. *Worldviews on Evidence-Based Nursing* 4; 126-145.

Greenhalgh T, Robert G, Macfarlane F, Bate P and Kyriakidou O 2004. Diffusion of innovations in service organizations: systematic review and recommendations. *Milbank Quarterly* 82; 581-629.

Humphries S, Stafinski T, Mumtaz Z and Menon D 2014. Barriers and facilitators to evidence-use in program management: a systematic review of the literature. *BMC Health Services Research* 14; 171.

Innvaer S, Vist G, Trommald M and Oxman A 2002. Health policy-makers' perceptions of their use of evidence: a systematic review. *Journal of Health Services Research & Policy* 7; 239-44.

Kajermo KN, Bostrom AM, Thompson DS, Hutchinson AM, Estabrooks CA and Wallin L 2010. The BARRIERS scale -- the barriers to research utilization scale: A systematic review. *Implementation Science* 5; 32.

Koon AD, Rao KD, Tran NT and Ghaffar A 2013. Embedding health policy and systems research into decision-making processes in low- and middle-income countries. *Health Research Policy and Systems* 11.

Lavis J, Davies H, Oxman A, Denis JL, Golden-Biddle K and Ferlie E 2005. Towards systematic reviews that inform health care management and policy-making. *Journal of Health Services & Research Policy* 10 Suppl 1; 35-48.

Lavis JN, Hammill AC, Gildiner A, McDonagh RJ, Wilson MG, Ross SE, et al. 2005. *A systematic review of the factors that influence the use of research evidence by public policymakers. Final report submitted to the Canadian Population Health Initiative.* Hamilton, Canada, McMaster University Program in Policy Decision-Making.

Liang Z, Howard P, Leggat S and Murphy G 2012. A framework to improve evidence-informed decision-making in health service management. *Australian Health Review* 36; 284-289.

Liverani M, Hawkins B and Parkhurst JO 2013. Political and institutional influences on the use of evidence in public health policy. A systematic review. *PLoS ONE [Electronic Resource]* 8; e77404.

MacGregor JC, Wathen N, Kothari A, Hundal PK and Naimi A 2014. Strategies to promote uptake and use of intimate partner violence and child maltreatment knowledge: an integrative review. *BMC Public Health* 14; 862.

McCormack B, Rycroft-Malone J, DeCorby K, Hutchinson AM, Bucknall T, Kent B, et al. 2013. A realist review of interventions and strategies to promote evidence-informed healthcare: A focus on change agency. *Implementation Science* 8; 107.

Mitton C, Adair CE, McKenzie E, Patten SB and Waye Perry B 2007. Knowledge transfer and exchange: review and synthesis of the literature. *Milbank Quarterly* 85; 729-768.

Moore G, Redman S, Haines M and Todd A 2011. What works to increase the use of research in population health policy and programmes: A review. *Evidence & Policy* 7; 277-305.

Moore G, Todd A and Redman S 2009. Strategies to increase the use of evidence from research in population health policy and programs: Evidence Check rapid reviews brokered by the Sax Institute (http://www.saxinstitute.org.au) for the Primary Health and Community Partnerships Unit, NSW Department of Health.

Oliver K, Innvar S, Lorenc T, Woodman J and Thomas J 2014. A systematic review of barriers to and facilitators of the use of evidence by policymakers. *BMC Health Services Research* 14; 2.

Orton L, Lloyd-Williams F, Taylor-Robinson D, O'Flaherty M and Capewell S 2011. The use of research evidence in public health decision making processes: systematic review. *PLoS ONE [Electronic Resource]* 6; e21704.

Sadeghi-Bazargani H, Tabrizi JS and Azami-Aghdash S 2014. Barriers to evidence-based medicine: a systematic review. *Journal of Evaluation in Clinical Practice* 20; 793-802.

Solomons NM and Spross JA 2011. Evidence-based practice barriers and facilitators from a continuous quality improvement perspective: an integrative review. *Journal of Nursing Management* 19; 109-20.

Squires JE, Estabrooks CA, Gustavsson P and Wallin L 2011. Individual determinants of research utilization by nurses: a systematic review update. *Implementation Science* 6; 1.

Wallace J, Byrne C and Clarke M 2012. Making evidence more wanted: a systematic review of facilitators to enhance the uptake of evidence from systematic reviews and meta-analyses. *International Journal of Evidence-Based Healthcare* 10; 338-46.

Wallace J, Nwosu B and Clarke M 2012. Barriers to the uptake of evidence from systematic reviews and meta-analyses: a systematic review of decision makers' perceptions. *BMJ Open* 2.

Williams I, McIver S, Moore D and Bryan S 2008. The use of economic evaluations in NHS decision-making: a review and empirical investigation. *Health Technology Assessment* 12; iii.

# Characteristics of included studies

Note: systematic reviews are grouped as: 1) practice only, 2) policy and practice, 3) policy only. Within groups, reviews are sorted alphabetically.

## Table 3a. Practice only (n=15)

| **Study** | **Objectives** | **Target population / Setting** | **Strategy tested / Domain** | **Outcomes reported** | **Date of last search** | **AMSTAR score** | **Included study designs & number** | **Country or region of studies** | **Results** |
| --- | --- | --- | --- | --- | --- | --- | --- | --- | --- |
| [Abdullah et al. (2014)](#_ENREF_1) | To determine the effectiveness of mentoring as a KT intervention designed to increase healthcare professionals’ use of evidence in clinical practice. | Healthcare professionals responsible for patient care /  Clinical care settings | Mentoring or Opinion leaders /  Pull Efforts | Knowledge use: instrumental, conceptual, and enablers of knowledge use;  Impact on patients, practitioners, and organization | December 2012 | 9 | 10 studies:  6 cluster-RCTs  1 CCT  1 CBA  2 pre- and post-test | 7 USA  2 Canada  1 Australia | Only one study, with low risk of bias, compared a multifaceted intervention with mentoring to the same kind of intervention without mentoring (i.e. educational meetings combined with educational materials, and audit and feedback). This study showed mixed effects for practitioners’ behavior, with one outcome improving and others showing no difference. The other nine studies with mentoring as part of a multifaceted intervention (compared to a single intervention without mentoring or to no intervention) showed various effects on practitioners, patients, and organizations, making it difficult to determine the added effect of mentoring. Further research is needed. |
| [Brettle et al. (2011)](#_ENREF_2) | 1. To build on previous models of clinical librarianship and determine which models of clinical librarian services have been evaluated. 2. To determine whose perspective has been evaluated when evaluating clinical librarian services. 3. To determine the quality of the methods used to evaluate clinical librarian services. 4. To determine what outcome measures have been used to evaluate clinical librarian services and establish their appropriateness. 5. To update previous reviews evaluating the effectiveness of clinical librarian services. | Healthcare professionals; managers making decisions about clinical care /  Clinical care settings | Clinical librarian services /  Facilitating Pull Efforts | Patient care, e.g. better informed decisions, diagnosis and change in  drug or therapy;  Saving health professionals time;  Cost saved;  Literature search results useful;  Improved information literacy skills;  Improved confidence | Autumn 2009 | 8 | 18 studies:  1 RCT  1 Before and after study, 16 Studies that used surveys, case studies and/or action research | 13 UK  4 USA  1 Netherlands | There are four clear models of clinical library service provision: 1) Question and answer service, 2) Outreach, 3) Question and answer service plus critical appraisal, and 4) Outreach plus critical appraisal and synthesis. CLs are effective in providing relevant and useful information and are perceived to save clinicians’ time. The majority of studies reported a positive impact on patient care; a quarter of studies identified a positive impact on the choice of drug or therapy. On the whole, however only a small number of studies were able or sought to quantify the impact made by the CL. The quality of clinical librarian studies is improving, but more work is needed on reducing bias and providing evidence of specific impacts on patient care. |
| [Dizon et al. (2012)](#_ENREF_6) | To systematically find, assess and synthesise the overall evidence for the effectiveness of evidence-based practice (EBP) training programs in improving the knowledge, skills, attitudes and behaviour of any allied health professional and, in  particular, the specific allied health disciplines from which this evidence was generated. We also aimed to establish evidence regarding the effectiveness of various components of EBP training programs. | Allied health professionals (allied, scientific and complementary health ) /  NS | Education in EBP /  Pull Efforts | Change in:  Knowledge, Skills,  Attitudes, and/or Behaviour  to EBP. | NS | 6 | 6 studies:  4 RCTs  2 before and after | 2 Australia  1 Hong Kong  2 UK  1 USA | Overall, there is limited research regarding training of allied health professionals in evidence-based  practice and learning outcomes. Knowledge and skills were influenced by any EBP training program (however, the most common approach was in the form of lectures and workshops), and significant change was achieved regardless of the type of outcome measure. EBP programs that utilised co-interventions such as opinion leaders resulted in improvements in attitudes. There was no clear evidence of which educational components influence EBP behaviours. Only the social work before and after study, which reassessed outcomes after 3 months, reported significant changes in attitudes and behaviours. Training took from 3 hours to 2 days. While there was information on training program components, there was no evidence of effectiveness related to learning outcomes. |
| [Flodgren et al. (2011a)](#_ENREF_8) | To assess the effectiveness of local opinion leaders in improving the behavior of healthcare professionals and patient outcomes. | Healthcare professionals in charge of patient care /  Clinical care settings | Local opinion leaders /  Pull Efforts | Compliance with desired practice;  (No patient outcomes reported). | May 2009 | 10 | 18 cluster-RCTs | 1 Argentina and Uruguay 6 Canada  1 China (Hong Kong)  10 USA | Opinion leaders alone or in combination with other interventions may successfully promote evidence-based practice, but effectiveness varies both within and between studies. These results are based on heterogeneous studies differing in terms of type of intervention, setting, and outcomes measured. The median adjusted risk difference (RD) for the main comparisons were: i) Opinion leaders compared to no intervention, +0.09; ii) Opinion leaders alone compared to a single intervention, +0.14; iii) Opinion leaders with one or more additional intervention(s) compared to the one or more additional intervention(s), +0.10; iv) Opinion leaders as part of multiple interventions compared to no intervention, +0.10. Overall, across all 18 studies the median adjusted RD was +0.12 representing a 12% absolute increase in compliance in the intervention group. |
| [Gagnon et al. (2010)](#_ENREF_10) | To review current evidence on the effectiveness of interventions promoting healthcare professionals’ clinical information retrieval technology (CIRT)* adoption and optimal use. | Healthcare practitioners providing clinical care to patients /  Clinical care settings | Access to CIRT and:  Educational meetings;  Educational materials;  Educational outreach visits;  Audit and feedback; or Financial disincentives /  Pull Efforts | Objective measures of:  CIRT adoption or use;  Quality of use;  General clinical performance or process outcomes. | March 2008 | 5 | 9 studies:  5 RCTs  4 cluster-RCTs | 4 USA  3 Canada  1 Hong Kong  1 Australia | Overall, CIRT capability (defined as the proportion of health care professionals capable of performing a search, successfully searching, or using CIRT) improved with intervention (OR=2.10; 95% CI; 1.63 to 2.71; p=0.0001), though there was significant heterogeneity in the studies. Subgroup analysis based on the type of interventions showed that educational meetings were the only type of interventions reporting consistent positive effects on CIRT capability (OR=3.65; 95% CI; 2.48 to 5.39; p=0.0001). However, there is no strong evidence against no effect for interventions providing educational material (OR=1.26, 95% CI; 0.85 to 1.87; p=0.24) or multifaceted interventions (OR=1.84, 95% CI; 0.85 to 3.58; p=0.07). In addition one study found that introducing user fees to access MEDLINE was found to significantly reduce the proportion of participants who conducted a search (relative difference=−35%, 95% CI; −57% to −13%; p=0.002). |
| [Giguere et al. (2012)](#_ENREF_12) | 1. To assess the effect of printed educational materials (PEMs) on the practice of healthcare professionals and patient health outcomes. 2. To explore the influence of some of the characteristics of the PEMs (e.g. source, content, format) on their effect on professional practice and patient outcomes. | Healthcare professionals /  Clinical care settings | Passive dissemination of PEMs (incl. clinical practice guidelines, monographs, and publications in peer-reviewed journals) - delivered personally or through mass mailing /  Push Efforts | Professional practice outcomes;  Patient outcomes. | June 2011 | 7 | 45 studies:  6 RCTs  8 cluster-RCTs  31 interrupted  time series | 1 Belgium  1 Brazil  12 Canada  1 Finland  1 Germany  1 Italy  2 Japan  2 Netherlands  1 Spain  11 UK  11 USA  1 Canada and USA  . | The results of this review suggest that when used alone and compared to no intervention, PEMs may have a small beneficial effect on professional practice outcomes. There is insufficient information to reliably estimate the effect of PEMs on patient outcomes. Based on seven RCTs and 54 outcomes, the median absolute risk difference in categorical practice outcomes was 0.02 when PEMs were compared to no intervention (range from 0 to +0.11). Based on three RCTs and eight outcomes, the median improvement in standardized mean difference for continuous professional practice outcomes was 0.13 when PEMs were compared to no intervention (range from -0.16 to +0.36). From the interrupted time series studies, we calculated improvements in professional practice outcomes across studies after PEM dissemination (standardized median change in level = 1.69). From the data gathered, we could not comment on which PEM characteristic influenced their effectiveness. |
| [Harris et al. (2008)](#_ENREF_13), [Harris et al. (2011)](#_ENREF_14) | The objective for this systematic review was to determine whether the Journal Club (JC) is an effective intervention in supporting evidence-based decision making in health professionals, and to explore whether it is possible to determine which elements of a JC contribute to effectiveness. | Healthcare professionals /  Educational settings;  Clinical care settings | Journal clubs /  Pull Efforts | Change in reading behavior;  Confidence in ability to critically appraise research;  Demonstrated knowledge and critical appraisal skills; Ability to apply findings to clinical practice. | February 2007 | 3 | 18 studies:  1 RCT  1 CCT  8 before and after  6 surveys  1 observational  1 case-control | 11 USA  ? Canada  ? England  ? Australia  ? Pakistan | The effectiveness of journal clubs in supporting evidence-based decision making is not clear because only seven studies attempted to measure this endpoint and they relied on self-report. Studies reported improvements in self-reported reading behavior (N=5/11), confidence in critical appraisal (N=7/7), critical appraisal test scores (N=5/7) and ability to use findings (N=5/7). No studies reported on patient outcomes. There is no ideal format for a JC. Groups need to tailor the club according to learner needs and level of training. Realist synthesis identified potentially ‘active educational ingredients’, including mentoring, brief training in clinical epidemiology, structured critical appraisal tools, adult-learning principles, multifaceted teaching approaches and integration of the journal club with other clinical and academic activities. |
| [Horsley et al. (2011)](#_ENREF_15) | To assess the effects of teaching critical appraisal skills to health professionals on the process of care, patient outcomes and knowledge of health professionals. | Healthcare professionals with direct patient care /  Clinical care settings | Education in critical appraisal /  Pull Efforts | (No patient outcomes or process of care outcomes reported);  Knowledge of health professionals. | June 2011 | 10 | 3 RCTs | 1 Canada  1 England  1 USA | Low-intensity critical appraisal teaching interventions in healthcare populations may result in modest gains. Improvements to research examining the effectiveness of interventions in healthcare populations are required; specifically rigorous randomized trials employing interventions using appropriate adult learning theories. Statistically significant improvements in participants’ knowledge were reported in domains of critical appraisal (variable approaches across studies) in two of the three studies. We determined risk of bias to be ‘unclear’ and as such considered this to be ‘plausible bias that raises some doubt about the results’. |
| [Ilic and Maloney (2014)](#_ENREF_16) | A systematic review was performed to identify what type of educational method is most effective at increasing medical trainees’ competency in evidence-based medicine (EBM). | Medical trainees (undergraduate or postgraduate)  Educational setting | Education in EBM /  Pull Efforts | Change in EBM competency as measured by knowledge, skills, attitudes or behavior towards EBM. | February 2013 | 6 | 9 RCTs:  2 RCTs compared education in EBM to a control group.  7 RCTs compared two different EBM interventions | 1 Australia  1 Canada  1 Hong Kong  1 Japan  1 Norway  2 UK  2 USA | Learner competency in EBM (knowledge, skills, attitudes or behavior towards EBM) increased post-intervention across all studies. However, it cannot be concluded that education in EBM improves competency in EBM – only two of the RCTs had a true control group. One RCT (by the author of the systematic review) had low risk of bias and found no difference between education in EBM and control ([Ilic et al. 2012](#_ENREF_17)). The other RCT did find a significant difference between intervention and control but it had an unclear risk of bias. No difference in learner outcomes was identified across a variety of educational modes, including lecture versus online, direct versus self-directed, multidisciplinary versus discipline-specific groups, lecture versus active small group facilitated learning. |
| [Li et al. (2009)](#_ENREF_19) | First, we examined how communities of practice (CoPs) were defined and used in the business and health sectors. Second, we assessed the evidence on the effectiveness of CoPs in health care settings for improving the uptake of best practices and mentoring new practitioners.  ***Note:*** *only the results of the second objective are presented here.* | Healthcare practitioners /  Clinical care settings | Communities of practice /  Pull Efforts | None | September 2005 | 3 | 0 primary studies on effectiveness | NA | We did not find any paper in the health sector that met the eligibility criteria for the quantitative analysis, and so the effectiveness of CoPs in this sector remained unclear. |
| [Lobach et al. (2012)](#_ENREF_20) | To catalogue study designs used to assess the clinical effectiveness of clinical decision support systems (CDSSs) and knowledge management systems (KMSs), to identify features that impact the success of CDSSs/KMSs, to document the impact of CDSSs/KMSs on outcomes, and to identify knowledge types that can be integrated into CDSSs/KMSs.  ***Note:*** *only the KMSs results are eligible for inclusion here. The CDSSs were not explicitly evidence-based.* | Healthcare providers /  Academic and community settings, clinics, practices, hospitals, and long-term care  facilities | Knowledge management systems^†^ /  Pull Efforts | Changes in:  Organization of health care delivery;  Workload and efficiency for the user;  Health care process measures;  Clinical outcomes. | December 2010 | 10 | 148 RCTs (CDSSs and KMSs), including  3 RCTs on KMSs | NS | Only 3 RCTs assessed the impact of KMSs on any outcomes. Evidence for the effectiveness of KMSs on any outcomes is minimal. |
| [McCormack et al. (2013b)](#_ENREF_22) | To provide information on how to best translate and disseminate research-based evidence to patients and clinicians in three separate areas: KQ1 - communicating evidence, KQ2 - disseminating evidence, and KQ3 - communicating uncertainty. | General public and patients; Clinicians /  Clinical care settings;  Community-based settings | Communication of evidence;  Dissemination of evidence;  Communicating uncertainty. /  Push Efforts | Knowledge outcomes;  Health-related  decisions and behavior outcomes;  Clinical outcomes. | March 2013 | 9 | 54 studies (61 articles)  Mostly RCTs and cluster-RCTs | NS | For KQ 1, we found that investigators frequently blend more than one communication strategy in interventions and the evidence was insufficient to make recommendations. For KQ 2, we found that, compared with single dissemination strategies, multicomponent dissemination strategies are more effective at enhancing clinician behavior, particularly for guideline adherence. Key findings for KQ 3 indicate that evidence on communicating overall strength of recommendation and precision was insufficient, but certain ways of communicating directness and net benefit may be helpful in reducing uncertainty. |
| [Perrier et al. (2011b)](#_ENREF_28) | To determine the impact on professional performance and health care outcomes of interventions for seeking, appraising, and applying evidence from systematic reviews (as a source document) in decision-making by clinicians. | Healthcare practitioners providing clinical care to patients /  Clinical care settings | Educational visit + access to Cochrane Library; Educational meetings;  Multifaceted + access to WHO Reproductive Health Library; Education +/- remuneration. /  Facilitating Pull Efforts, Pull Efforts | Objective measure of professional performance behavior | July 2009 | 9 | 5 cluster-RCTs | 1 Canada  1 Mexico and Thailand  1 Argentina and Uruguay  2 UK | One study reported positive outcomes in improving preventive care. Three studies focused on obstetrical care, with two reporting no impact on professional practice change, and one study reporting increases in the use of prophylactic oxytocin and episiotomy. One study found no improvement in the sealant rate of newly erupted molars among dentists in Scotland. No studies were identified that examined health outcomes for patients. Overall, there is insufficient evidence to support or refute interventions for seeking, appraising, and applying evidence from systematic reviews in decision-making by clinicians. |
| [Worbes-Cerezo et al. (2010)](#_ENREF_30) | In this systematic review we synthesized scientific evidence on the cost-effectiveness of knowledge translation methods in health professionals.  ***Note:*** *only the abstract is available. An AMSTAR assessment could not be done.* | Health professionals /  NS | Education, academic detailing, software support, or combinations /  Pull Efforts | NS | March 2010 | - | 18 economic evaluations | NS | Most of the interventions are effective in knowledge translation, i.e. fulfil the function for which they have been designed. However, most of them are relatively costly, and therefore less cost-effective. |
| [Yamada et al. (2015)](#_ENREF_31) | The aim of this systematic review was to identify and evaluate the effectiveness of toolkits as a KT strategy for facilitating the implementation of evidence into clinical care and to inform future development, implementation and evaluation of toolkits.  Toolkits include multiple resources (KT tools and strategies) for educating and/or facilitating behavior change. | Health professionals, patients, and caregivers /  Healthcare settings | Toolkit – multiple resources (tools and strategies);  Toolkit embedded in multi-strategy intervention /  Climate, Push Efforts, Pull Efforts | Clinical outcomes;  Implementation outcomes. | November 2013 | 6 | 39 studies of varying designs, 8 were rated as moderate to strong:  3 RCT  4 cluster-RCTs  1 CCT | 2 Canada  1 Italy  5 USA | The types of resources embedded within toolkits varied but included predominantly educational materials. Three of the eight studies evaluated the toolkit as a single KT intervention, while five embedded the toolkit into a multi-strategy intervention. Only four of five multi-strategy intervention studies demonstrated partial to mostly effective results. Of the three single KT intervention studies, two were mostly effective at changing clinical outcomes. No studies evaluated the relative effectiveness of each KT strategy (e.g. use of audit and feedback); therefore, it was not possible to determine which components contributed to the change in outcomes. |

## Table 3b. Policy & Practice (n=7)

| **Study** | **Objectives** | **Target population / Setting** | **Strategy tested / Domain** | **Outcomes reported** | **Date of last search** | **AMSTAR score** | **Included study designs & number** | **Country or region of studies** | **Results** |
| --- | --- | --- | --- | --- | --- | --- | --- | --- | --- |
| [Flodgren et al. (2011b)](#_ENREF_9) | To evaluate the effectiveness of external inspection of compliance with standards in improving healthcare organization behavior, healthcare professional behavior and patient outcomes. | Health care managers and professionals /  Hospitals, other health care organizations (primary or community-based). | External inspection of compliance with standards /  Climate | Compliance with accreditation standards;  Methicillin-resistant *Staphylococcus aureus* (MRSA) infection rate | May 2011 | 9 | 2 studies:  1 cluster-RCT  1 before and after | 1 England  1 South Africa | We only identified two studies for inclusion in this review, which highlights the paucity of high-quality controlled evaluations of the effectiveness of external inspection systems. No firm conclusions could therefore be drawn about the effectiveness of external inspection on compliance with standards. The cluster-RCT showed mixed effects of a hospital accreditation system on the compliance with COHSASA (the Council for Health Services Accreditation for South Africa) accreditation standards and eight indicators of hospital quality. For the before and after study, re-analysis of the MRSA data (as an interrupted time series study) showed statistically non-significant effects of the Healthcare Commissions Infection Inspection program. |
| [Gifford et al. (2007)](#_ENREF_11) | 1. To describe leadership activities of nurse managers that influence nurses’ use of research evidence; and 2. To identify interventions aimed at supporting nurse managers to influence research use in clinical nursing practice.   ***Note:*** *only results related to the second objective are presented here.* | Nurse managers and administrators with aim to change use of research in nurse practitioners /  Clinical care settings including hospital, community, home, long-term care. | Nurse manager education and/or support; change in policy, plans, structures; establishment of networks; leadership /  Climate, Push Efforts, Pull Efforts | Process of care;  Patient outcomes;  Research use | January 2006 | 4 | 12 studies of which 4 assessed interventions:  1 RCT  2 before and after  1 case study with survey | 1 Australia  1 Canada  2 USA | Four of the eight quantitative studies included evaluations of the effect of an intervention that included managers to promote nurses’ use of research evidence. Three studies showed positive results (2 before and after studies, 1 case study with a survey); however, lack of a control group makes it impossible to draw firm conclusions about the effects of these interventions. The RCT found no effect on selected patient outcomes. It would appear that leadership development at multiple levels is important to address the complexity of factors involved in nurses’ use of research, and should be a focus for future research. |
| [LaRocca et al. (2012)](#_ENREF_18) | To identify the effectiveness of KT strategies used to promote evidence-informed decision making among public health decision makers. | Public health and health promotion decision makers (practitioners, managers and policy makers) /  Public health or community setting | Communities of practice (CoPs);  Education;  Education + information service + free access to databases;  Dissemination of information;  Access to SRs +/- tailored messaging +/- knowledge brokers.  Push Efforts, Facilitating Pull Efforts, Pull Efforts, Linkage and Exchange | Change in practice;  Change in knowledge.  (Change in skill outcomes not reported). | April 2010 | 7 | 5 studies:  4 RCTs  1 interrupted time series | 2 Canada  1 England  1 Norway  1 USA | For CoPs there was no significant change in knowledge or practice. Dissemination of information via a printed pamphlet, CD-ROM or internet led to a significant improvement in knowledge but no change in practice. The multifaceted intervention that included education, a question and answer service and free access to databases lead to a significant improvement in knowledge but no change in practice. The intervention by Dobbins and colleagues ([Dobbins et al. 2009](#_ENREF_7)), which combined access to SRs with tailored and targeted messaging, was the only one to lead to a change in practice. The educational session was insufficient to significantly change adherence to a national suicide prevention guideline. Simple or single KT strategies were shown in some circumstances to be as effective as complex, multifaceted ones when changing practice including tailored and targeted messaging. |
| [McCormack et al. (2013a)](#_ENREF_21) | This review addresses the following question: What change agency characteristics work, for whom do they work, in what circumstances and why? ‘Change agency’ is defined as ‘organization or other unit that promotes and supports adoption and implementation of innovations’. Facilitators, knowledge brokers and opinion leaders are examples of change agency strategies used to promote knowledge utilization. | Healthcare practitioners and policymakers /  NS | Opinion leaders, facilitators (internal/external), practice developers, education outreach, academic detailing, and the use of multiple change agents /  Pull Efforts, Linkage and Exchange | NS | March 2007 | 2 | 52 studies  Study types not limited or specified. | NS | 52 articles were included (study types not limited or specified). Our review suggests that change agents who are adequately supported and resourced (context) and who model the roles and practices they espouse (mechanism), have greater potential to achieve evidence-informed healthcare (outcome). While evidence of effectiveness is weak, in some cases in terms of outcomes data, there is evidence that supports the importance of opinion leader and facilitator roles. More detailed description of interventions, outcome measures, the context, intensity, and levels at which interventions are implemented are required in order to understand how change agent interventions effect evidence-informed health care. |
| [Mitton et al. (2007)](#_ENREF_23) | Our primary aim for this review was to examine and summarize the current evidence base for knowledge translation and exchange (KTE) in relation to health policy, resulting in an evidence-based resource for planning KTE processes.  ***Note:*** *63 higher-quality papers were included, of which 10 were implementation studies. Only the results of the implementation studies are presented here.* | Policy and decision makers (not clinicians) | Face-to-face exchange;  Education;  Networks and CoPs;  Facilitated meetings b/n decision makers and researchers; Web-based information and communication. /  Push Efforts, Facilitating Pull Efforts, Pull Efforts, Linkage and Exchange | NS | January 2006 | 5 | 10 studies  1 Post-test with control group  2 Single group post-test  2 Multiple case study  1 Parallel case study  4 Case study or report | 8 Canada  2 UK | In short, based on these studies, we did not find an “off the shelf” set of recommendations for developing and implementing KTE strategies. This difficulty is due in part to the relatively small number of implementation studies across fields in health care and also to the even less formal and/or rigorous evaluation of these strategies. At this time there is an inadequate evidence base for doing “evidence-based” KTE for health policy decision making. |
| [Murthy et al. (2012)](#_ENREF_26) | To identify and assess the effects of information products based on the findings of systematic review evidence and organizational supports and processes designed to support the uptake of systematic review evidence by health system managers, policy makers and healthcare professionals. | Health system managers, policy makers and healthcare professionals /  Clinical care settings, a government public health department | Multifaceted + access to WHO Reproductive Health Library; Educational visit + access to Cochrane Library; Dissemination of summaries of SRs;  Access to SRs +/- tailored messaging +/- knowledge brokers;  Analgesic league table (based on SRs) + audit and feedback + education; Summary of findings table in SRs  Push Efforts, Facilitating Pull Efforts, Linkage and Exchange | Measures of research utilization:  Clinical practice; Changes in clinician behavior; Healthcare management decisions. | March 2011 (full search)  March 2012 (Medline only) | 9 | 8 studies*:*  3 RCTs  2 cluster RCTs  3 interrupted times series | 1 Canada  5 UK  1 Mexico and Thailand  1 NS | The overall quality of the evidence was very low to moderate. Mass mailing a printed bulletin which summarizes systematic review evidence may improve evidence-based practice when there is a single clear message, if the change is relatively simple to accomplish, and there is a growing awareness by users of the evidence that a change in practice is required (3 interrupted time series studies). There is insufficient evidence to support the other multifaceted interventions (4 RCTs). Summary of findings tables in Cochrane Reviews lead to increased acceptability (1 RCT). |
| [Wallace et al. (2014)](#_ENREF_29) | To identify interventions to enhance evidence uptake from systematic reviews, meta-analyses and the databases containing them. | All decision makers, including doctors, nurses, policymakers, the public and patients /  Hospitals, a government public health department, and academic settings | Educational visits;  Education on EBM;  Summaries of SR;  Manual of  Cochrane SRs;  Access to SRs +/- tailored messaging +/- knowledge brokers. /  Push Efforts, Facilitating Pull Efforts, Pull Efforts, Linkage and Exchange | Knowledge;  Attitudes;  Decision maker behavior (research use);  Practice change;  Quality of life | January 2014 | 8 | 10 studies:  5 RCTs  3 cluster-RCTs  1 CCT  1 before and after | 1 Australia  1 Canada  4 UK  1 USA  1 Germany, Hungary,  Spain, Switzerland and the UK. 1 UK and Netherlands 1 Mexico and Thailand | Three studies of low-to-moderate risk of bias, identified interventions that showed a statistically significant improvement: educational visits, short summaries of systematic reviews, and tailored and targeted messaging. Promising interventions include e-learning, computer-based learning, interactive workshops, use of knowledge brokers and an e-registry of reviews but these interventions need to be developed further. New strategies are required to encompass neglected barriers and facilitators. |

##

## Table 3c. Policy only (n=5)

| **Study** | **Objectives** | **Target population / Setting** | **Strategy tested / Domain** | **Outcomes reported** | **Date of last search** | **AMSTAR score** | **Included study designs & number** | **Country or region of studies** | **Results** |
| --- | --- | --- | --- | --- | --- | --- | --- | --- | --- |
| [Bunn and Sworn (2011)](#_ENREF_3) | The aim of this review was to identify and evaluate potential strategies for increasing the impact of systematic reviews on policy. | Health policy decision makers /  Public health departments or units | Collaborative approaches b/n researchers and decision makers;  Collaborative approaches + SRs;  Dissemination strategies. /  Push Efforts; Linkage and Exchange | NS | January 2011 | 6 | 11 studies (13 papers):  1 RCT  4 Surveys  2 Qualitative  4 Descriptive or discussion papers | 7 Canada  1 Iran  2 UK  1 USA | Only one included study was a formal evaluation – an RCT by Dobbins and colleagues ([2009](#_ENREF_7)). It found that:   - having access to a registry of synthesized and translated research evidence (control group) has no impact on evidence-informed decision making (EIDM) (p<0.45); - targeted messaging significantly more effective in promoting EIDM than other strategies (p<.009); - simple KT and exchange strategies may be as effective as complex ones (but need to be active rather than passive); - knowledge brokering (KB) was more effective in those organizations that placed less value on research evidence and was less effective in those organizations that already recognized the importance of evidence-based decision making.   Qualitative findings contradicted quantitative results.  Participants in the KB group perceived KB to have significantly affected EIDM capacity for them personally as well as for their organization.  Authors concluded that KB intervention may not have contained all the necessary components to produce a positive effect.  More work is needed to evaluate the benefits of the different strategies. |
| [Chambers et al. (2011)](#_ENREF_4) | To perform a systematic scoping review of knowledge-translation resources and evaluations of them. These resources include summaries of SRs, overviews of SRs, and policy briefs based on SRs.  ***Note:*** *only the evaluations of the resources are eligible for inclusion here.* | Healthcare policy makers at the national or local level /  National or local government or health authorities | Packaging of systematic reviews as:  Summaries; Overviews;  Policy briefs. /  Push efforts; Facilitating User Pull | Perceived usefulness of the resource;  Use of the resource. | October 2009 | 4 | 7 evaluation studies:  4 Surveys of users  1 Interviews in development stage  2 Descriptive | 1 Australia  1 Canada  1 South Africa  3 UK  1 Low and middle-income countries | Twenty knowledge-translation resources were identified and seven evaluation studies were included. No studies evaluated policy briefs. The quality of evidence is very low. The majority of studies reported on the perceived usefulness of the service, although there were some examples of review-based resources being used to assist actual decision making. However, the extent to which these resources are used and are found useful by policymakers is unclear. More evaluations of these resources are required to ensure that users’ needs are being met, to demonstrate their impact, and to justify their funding. |
| [Clar et al. (2011)](#_ENREF_5) | The primary objective of this review was to assess the effects of interventions to improve the uptake of research into health policies in low and middle-income countries. A secondary objective was to identify the barriers and facilitators to the uptake of research evidence derived from intervention and non-intervention studies.  ***Note:*** *only the results of the intervention component are included here.* | Health policy makers at a local, sub-national, national or global level /  Low and middle-income countries | Complex, multifaceted – Almost all of the studies included two main sub-components: local research and extensive stakeholder involvement or collaboration with stakeholders. /  Multiple domains, including Linkage and Exchange | Change in health policies based on evidence uptake (primary outcome);  Other policy-related outcomes; Practice-related outcomes;  Health outcomes. | NS  (But hand searching conducted October 2010) | 7 | 21 reports of 25 interventions:  1 RCT  24 Qualitative | Large range of countries included but results not summarized in report:  12 Low income countries  2 Low and middle-income countries  11 Middle-income countries | All interventions had some positive effects in terms of policy-related outcomes. However, most of the intervention studies were descriptive case-studies with inadequate detail on methodology and intervention design. Thus, it is not possible from the data provided to draw any conclusions linking intervention sub-components to size of effect. However, the findings are broadly consistent with the findings from high-income countries on the need for multi-faceted, tailored interventions and on the importance of contextual influences, particularly organizational.  The most frequently cited components of interventions reporting positive effects on policy development included carrying out local research (e.g. for contextualization), ensuring intensive stakeholder engagement and collaboration, including training and capacity-building activities, and fostering community participation. (Note: these elements were also identified in the non-intervention studies as common factors in the analysis of barriers and facilitators to evidence uptake). |
| [Moore et al. (2011)](#_ENREF_24), [Moore et al. (2009)](#_ENREF_25) | The primary aim of the review was to analyze what is known about the extent to which strategies to increase the use of research in population health policies and programs are effective.  ***Note:*** *only the results of the 5 intervention studies are included here.* | Health policy makers; program managers /  Government public health department or unit; Health services; | Access to SRs +/- tailored messaging +/- knowledge brokers; Dissemination of SRs;  Interaction between users and producers of research;  Education;  Push Efforts, Facilitating Pull Efforts, Pull Efforts, Linkage & Exchange | Use of research in a policy or program;  Changes in  knowledge, attitudes, skills and behavior;  Opportunities to use research in collaboration with others. | September 2009 | 2 | 5 intervention studies:  1 RCT  1 cluster-RCT  1 matched case control  1 Post-intervention survey  1 Self-assessment pre and post-intervention | 4 Canada  1 UK | In interpreting the findings of these studies, the methodological problems must be borne in mind. The studies included in this review provide some evidence that the use of tailored targeted messages, with access to registries of research, may increase the use of research in policy development ([Dobbins et al. 2009](#_ENREF_7)). No study provided evidence that interaction between researchers and policy makers has an impact on the use of research. Training in the appraisal of research and its use appears to increase participants’ skills in critical appraisal and possibly their perceptions about the value of research, but not their use of research. The single study that examined the use of knowledge brokers did not show them to be effective ([Dobbins et al. 2009](#_ENREF_7)). |
| [Perrier et al. (2011a)](#_ENREF_27) | To determine the impact on professional performance and healthcare outcomes of interventions for seeking, appraising, and applying evidence from systematic reviews in decision making by health policymakers. | Health policy makers and Managers / | Dissemination of SRs; website +/- tailored messages +/- knowledge broker.  Push Efforts, Facilitating Pull Efforts; Linkage and Exchange | Use of research in a policy or program. | April 2010 | 8 | 2 studies (4 papers):  1 RCT  1 Post-intervention surveys at 3 months and 2 years | 2 Canada | Three articles described one study where five SRs were mailed to public health officials and followed up with surveys at three months and two years. The articles reported from 23% to 63% of respondents declaring they had used SRs in policymaking decisions. One RCT indicated that tailored messages combined with access to a registry of systematic reviews had a significant effect on policies made in the area of healthy body weight promotion in health departments but not on global evidence-informed decision making (p < 0.45), which was the primary outcome ([Dobbins et al. 2009](#_ENREF_7)). However, for the group that worked with a knowledge broker, 30% of participants had limited or no engagement with the knowledge broker, thus the authors recommend caution with the generalizability of these results. |

CBA – controlled before and after study; CCT – controlled clinical trial; CDSSs – clinical decision support systems; KT – knowledge translation; NA – not applicable; NS – not specified.

***** CIRT refers to databases, e.g. MEDLINE, and search engines that clinicians can use to retrieve general information on disease prevention, health promotion, diagnosis, treatment and prognosis when needed in order to answer clinical questions at the point-of-care.

^†^ Examples of KMSs include: information retrieval tools and knowledge resources that consist of distilled primary literature on evidence-based practices.

##

# Quality assessment of included systematic reviews

## Table 4a. Assessment of the included systematic reviews against the AMSTAR criteria – Practice only

| **No.** | **AMSTAR QUESTIONS** | **Abdullah et al. (2014)** | **Brettle et al. (2011)** | **Dizon et al. (2012)** | **Flodgren et al. (2011a)** | **Gagnon et al. (2010)** | **Giguere et al. (2012)** | **Harris et al. (2008), Harris et al. (2011)** |
| --- | --- | --- | --- | --- | --- | --- | --- | --- |
| 1 | Was an ‘a priori’ design provided? | Yes | Yes | Yes | Yes | Can't answer | Yes | Can't answer |
| 2 | Was there duplicate study selection and data extraction? | Yes | Yes | Can't answer | Yes | Yes | Yes | Yes |
| 3 | Was a comprehensive literature search performed? | Yes | Yes | Yes | Yes | No | Yes | Yes |
| 4 | Was the status of publication (i.e. grey literature) used as an inclusion criterion? | Yes | Yes | Yes | Yes | Can't answer | No | Can't answer |
| 5 | Was a list of studies (included and excluded) provided? | Yes | No | No | Yes | No | Yes | No |
| 6 | Were the characteristics of the included studies provided? | Yes | Yes | Yes | Yes | Yes | Yes | No |
| 7 | Was the scientific quality of the included studies assessed and documented? | Yes | Yes | Yes | Yes | Yes | Yes | No |
| 8 | Was the scientific quality of the included studies used appropriately in formulating conclusions? | Yes | Yes | Yes | Yes | Yes | Yes | Not applicable |
| 9 | Were the methods used to combine the findings of studies appropriate? | Yes | Yes | No | Yes | Yes | No | Yes |
| 10 | Was the likelihood of publication bias assessed? | No | No | No | Yes | No | No | No |
| 11 | Was the conflict of interest stated? | No | No | No | No | No | No | No |
|  | **Total number of 'yes' scores** | **9** | **8** | **6** | **10** | **5** | **7** | **3** |

## Table 4b. Assessment of the included systematic reviews against the AMSTAR criteria – Practice only

| **No.** | **AMSTAR QUESTIONS** | **Horsley et al. (2011)** | **Ilic and Maloney (2014)** | **Li et al. (2009)** | **Lobach et al. (2012)** | **McCormack et al. (2013b)** | **Perrier et al. (2011b)** | **Yamada et al. (2015)** |
| --- | --- | --- | --- | --- | --- | --- | --- | --- |
| 1 | Was an ‘a priori’ design provided? | Yes | Can't answer | Can't answer | Yes | Yes | Yes | Can't answer |
| 2 | Was there duplicate study selection and data extraction? | Yes | Yes | Yes | Yes | Yes | Yes | Yes |
| 3 | Was a comprehensive literature search performed? | Yes | No | Yes | Yes | Yes | Yes | Yes |
| 4 | Was the status of publication (i.e. grey literature) used as an inclusion criterion? | Yes | No | Can't answer | No | No | Yes | No |
| 5 | Was a list of studies (included and excluded) provided? | No | Yes | No | Yes | Yes | Yes | No |
| 6 | Were the characteristics of the included studies provided? | Yes | Yes | No | Yes | Yes | Yes | Yes |
| 7 | Was the scientific quality of the included studies assessed and documented? | Yes | Yes | No | Yes | Yes | Yes | Yes |
| 8 | Was the scientific quality of the included studies used appropriately in formulating conclusions? | Yes | Yes | Not applicable | Yes | Yes | Yes | Yes |
| 9 | Were the methods used to combine the findings of studies appropriate? | Yes | Yes | Yes | Yes | Yes | Yes | Yes |
| 10 | Was the likelihood of publication bias assessed? | Yes | No | No | Yes | No | No | No |
| 11 | Was the conflict of interest stated? | Yes | No | No | Yes | Yes | No | No |
|  | **Total number of 'yes' scores** | **10** | **6** | **3** | **10** | **9** | **9** | **6** |

**Note:** an AMSTAR quality assessment could not be done on Worbes-Cerezo et al (2010) as only the abstract was available.

## Table 5. Assessment of the included systematic reviews against the AMSTAR criteria – Policy & Practice

| **No.** | **AMSTAR QUESTIONS** | **Flodgren et al. (2011b)** | **Gifford et al. (2007)** | **LaRocca et al. (2012)** | **McCormack et al. (2013a)** | **Mitton et al. (2007)** | **Murthy et al. (2012)** | **Wallace et al. (2014)** |
| --- | --- | --- | --- | --- | --- | --- | --- | --- |
| 1 | Was an ‘a priori’ design provided? | Yes | Can't answer | Can't answer | Can't answer | Can't answer | Yes | Yes |
| 2 | Was there duplicate study selection and data extraction? | Yes | No | Yes | Can't answer | Yes | Yes | Yes |
| 3 | Was a comprehensive literature search performed? | Yes | Yes | Yes | Yes | No | Yes | Yes |
| 4 | Was the status of publication (i.e. grey literature) used as an inclusion criterion? | Yes | No | Yes | No | Yes | Yes | Yes |
| 5 | Was a list of studies (included and excluded) provided? | Yes | No | No | No | No | Yes | No |
| 6 | Were the characteristics of the included studies provided? | Yes | Yes | Yes | No | Yes | Yes | Yes |
| 7 | Was the scientific quality of the included studies assessed and documented? | Yes | Yes | Yes | No | Yes | Yes | Yes |
| 8 | Was the scientific quality of the included studies used appropriately in formulating conclusions? | Yes | Yes | Yes | Not applicable | Yes | Yes | Yes |
| 9 | Were the methods used to combine the findings of studies appropriate? | No | No | Yes | Yes | No | Yes | Yes |
| 10 | Was the likelihood of publication bias assessed? | Yes | No | No | No | No | No | No |
| 11 | Was the conflict of interest stated? | No | No | No | No | No | No | No |
|  | **Total number of 'yes' scores** | **9** | **4** | **7** | **2** | **5** | **9** | **8** |

## Table 6. Assessment of the included systematic reviews against the AMSTAR criteria – Policy only

| **No.** | **AMSTAR QUESTIONS** | **Bunn and Sworn (2011)** | **Chambers et al. (2011)** | **Clar et al. (2011)** | **Moore et al. (2011), Moore et al. (2009)** | **Perrier et al. (2011a)** |
| --- | --- | --- | --- | --- | --- | --- |
| 1 | Was an ‘a priori’ design provided? | Can't answer | Yes | No | Can't answer | Yes |
| 2 | Was there duplicate study selection and data extraction? | Yes | Can't answer | No | Can't answer | Yes |
| 3 | Was a comprehensive literature search performed? | Yes | Yes | Yes | No | Yes |
| 4 | Was the status of publication (i.e. grey literature) used as an inclusion criterion? | Yes | Yes | Yes | No | Yes |
| 5 | Was a list of studies (included and excluded) provided? | No | No | Yes | No | No |
| 6 | Were the characteristics of the included studies provided? | Yes | Yes | Yes | Yes | Yes |
| 7 | Was the scientific quality of the included studies assessed and documented? | Yes | No | Yes | No | Yes |
| 8 | Was the scientific quality of the included studies used appropriately in formulating conclusions? | Yes | Not applicable | Yes | Not applicable | Yes |
| 9 | Were the methods used to combine the findings of studies appropriate? | No | No | Yes | No | Yes |
| 10 | Was the likelihood of publication bias assessed? | No | No | No | No | No |
| 11 | Was the conflict of interest stated? | No | No | No | Yes | No |
|  | **Total number of 'yes' scores** | **6** | **4** | **7** | **2** | **8** |

# Shortcuts taken in this review to make it rapid and AMSTAR quality assessment

## Shortcuts taken

- One reviewer screened titles and abstracts
- One reviewer extracted data with checking by a second reviewer
- Data extraction limited to key characteristics and results
- Data not extracted from all included systematic reviews, e.g. systematic reviews that were specific to a single health issue or to a single profession. Further, where there were multiple systematic reviews addressing the same intervention or question, data were only extracted from the most recent good quality review(s).
- Limit placed on language of publication – English, French, Portuguese and Spanish
- Narrow time frame – studies published from 2004
- Narrative synthesis only, although meta-analysis was not possible due to heterogeneity of included studies
- Publication bias not assessed, although no clear methods available for assessing publication bias qualitatively
- External peer review of report to funder not obtained

## Table 7. Assessment of this rapid review against the AMSTAR criteria

| **No.** | **AMSTAR questions** | **Assessment** |
| --- | --- | --- |
| 1 | Was an ‘a priori’ design provided? | Yes |
| 2 | Was there duplicate study selection and data extraction? | Yes |
| 3 | Was a comprehensive literature search performed? | Yes |
| 4 | Was the status of publication (i.e. grey literature) used as an inclusion criterion? | Yes |
| 5 | Was a list of studies (included and excluded) provided? | Yes |
| 6 | Were the characteristics of the included studies provided? | Yes |
| 7 | Was the scientific quality of the included studies assessed and documented? | Yes |
| 8 | Was the scientific quality of the included studies used appropriately in formulating conclusions? | Yes |
| 9 | Were the methods used to combine the findings of studies appropriate? | Yes |
| 10 | Was the likelihood of publication bias assessed? | No |
| 11 | Was the conflict of interest stated? | Yes |
|  | **Total number of 'yes' scores** | **10** |

1. The search of Health Systems Evidence was conducted by Kaelan Moat, McMaster Health Forum, McMaster University, to enable all relevant records to be viewed and imported into the EndNote database. [↑](#footnote-ref-1)
