# Supplementary material for: Designing a rapid response program to support evidence-informed decision-making in the Americas region: using the best available evidence and case studies
Source: Implement Sci. 2016 Aug 18;11:117. doi: 10.1186/s13012-016-0472-9 (PMC4990866; doi:10.1186/s13012-016-0472-9)
Supplement: Supplementary file 3 — Case studies. (DOCX 40 kb) [file 13012_2016_472_MOESM3_ESM.docx]

# Additional file 3. Case studies

## Questions used to guide the structured interviews

1. How did the service start?
2. Who is the service for (geographic scale, target organizations, target professions)?
3. How, and by whom, is the service funded?
4. Who pays for individual reviews? If ‘user-pays’ does this slow the process down?
5. Can you describe the governance of the service (key players, decision-making process)?
6. Do you use priority setting to determine which reviews to undertake? If so, what are the criteria?
7. How long does it take to conduct a review from start to finish (including organization of contracts/agreements if applicable)?
8. Do you use a standard method or protocol for conducting the reviews? Can you send me a copy? If not, how are the methods for each review decided?
9. Do you use a template for the final review? Can you send me a copy and/or an example review? Do you use any special software to produce the review?
10. Are completed reviews made publicly available? If not, why not? If so, where (e.g. website, repository) and how?
11. Can you tell me about the people that conduct the reviews – who they are, how you find them, how they are funded (e.g. permanent staff, contracted for each review), what skills they have?
12. Was it (is it) difficult to find adequately skilled people to conduct the reviews? Do you train them yourself?
13. What other ‘knowledge translation’ strategies do you conduct as part of the service to increase the chances that the reviews are used for policy and/or practice? Can you give me some examples?
14. Do you (or have you) conduct monitoring and evaluation of the service? If so, can you share any methods, results and/or publications related to this?
15. What do you see as the main ‘success factors’ for the service?
16. What are the biggest issues / problems that we should be aware of (past, present)?
17. What could be improved?
18. Is there anything else we should be aware of in relation to rapid review service/s that hasn’t already been covered?

## Cochrane Response Rapid Reviews by Cochrane Innovations

Cochrane Response Rapid Reviews are one of the products offered by Cochrane Innovations, the business arm of the Cochrane Collaboration, which was set up in part, in order to generate income for the Collaboration. In 2012-2013, it became a newly established program of the Cochrane Collaboration with the primary goal of providing a mechanism by which the Collaboration can respond to requests for commissioned reviews. At the time of its inception, a small global consortium of ‘Cochranites’ with an interest in rapid reviews had been assembled to develop materials to assist in the conduct of Cochrane Responses – which relied heavily on the Knowledge to Action (KTA) approach of the Ottawa Hospital Research Institute (OHRI), Canada <http://www.ohri.ca/kta/> [1]. Authors of Cochrane Responses were to be experienced Cochrane contributors. Each Response would require at least two reviewers, one information retrieval specialist, content expertise, and the necessary input of the client. The process added in 24-hour peer review periods with access to input from across the many content and methods experts that make up the Collaboration (which at the time was Cochrane’s 55 Review Groups, 14 Fields, and 12 Methods Groups). Each rapid review was to be placed on an 8-week schedule with intermediate deliverables scheduled throughout this period.

The first rapid review was produced by the Knowledge Synthesis Group (under the leadership of Dr. David Moher) at the Ottawa Hospital Research Institute (OHRI) team in 2013 and took 10 weeks to conduct. No further Cochrane Rapid Responses have been commissioned since. Reviews are conducted on a user-pays basis with the need for a contract/agreement as determined by Cochrane Innovations. The service is potentially available to any health-related organization in the world. A draft procedures manual is available to those active within the Collaboration to guide the rapid reviews but methods will vary, depending on the topic. Completed reviews are published on the Cochrane Innovations website.

**Other documents available to guide the reviews are:**

- A PICOTS Framework (Population, Intervention/Exposure, Comparison, Outcomes, Timing, Study Designs) – to help in formulation of the question and inclusion criteria
- Rapid Review ‘Evidence Summary’ Proposal [Template] – to guide the preparation of a protocol.
- A template for reporting – see the completed review for an example report.

**Strengths:**

Strengths of the program include the support of the Cochrane Collaboration, which includes its expertise and experience in conducting full systematic reviews.

**Challenges:**

It is very important to spend time with the funder of the review at the beginning to:

1. Ensure that they understand the product they will receive at the end.
2. Get the question right so that it meets the funder’s needs and can be answered within the time and resources available.

**Future work:**

To publish rapid reviews in the Cochrane Library.

**Sources:**

Chantelle Garrity, Senior Operations Manager, Ottawa Methods Centre, Ottawa Hospital Research Institute (OHRI), Ottawa, Canada – was involved in the first completed review.

Website: <http://innovations.cochrane.org/response>; <http://innovations.cochrane.org/about>

## McMaster Health Forum Rapid Response Program – McMaster University

The McMaster Health Forum’s Rapid Response Program provides responses to urgent requests from Canadian policymakers and stakeholders for evidence about health-system challenges based on a systematic search for information about problems, options and/or implementation considerations. The service can be requested in a three-, 10- or 30-day time frame, with details of the product provided detailed in a table on their website. Current funding from the Ontario Ministry of Health and Long-Term Care through a Health System Research Fund grant allows the Forum’s Rapid Response Program to conduct four rapid syntheses per year for policymakers and stakeholders in Ontario. For policymakers and stakeholders in other parts of Canada, requests can be taken on a user-pays basis. At this stage a prioritization process has not been necessary and all relevant reviews are accepted.

Rapid syntheses produced by the Forum focus on searching for relevant systematic reviews and economic evaluations using key databases (e.g. Health Systems Evidence), as well as for primary research when evidence from reviews is limited. Syntheses conducted in 30-business days provide a detailed synthesis of the key findings from systematic reviews and economic evaluations (and primary research where needed). This is supplemented with appendices that outline the key findings from each included document, quality assessments of the systematic reviews, and the countries in which studies included in the reviews were conducted. The rapid synthesis is also merit reviewed within the 30-business day timeline.

The syntheses are prepared by a team of staff with skills in systematic reviews and/or policy analysis with the support of a research assistant. Generally, one reviewer assesses the search results and conducts data extraction for each of these products, and quality appraisals of included reviews are extracted from Health Systems Evidence where the appraisals are conducted by two independent reviewers. All completed reviews are published on the program’s website and disseminated widely, including through social media. A briefing to the requestor can also be completed where needed. Eight completed reviews are available on the forum website and others will be published as they are completed, with more posted as they are completed.

**Documents available to guide the reviews are:**

- Summary of service timelines – what can be done in 3, 10 and 30 business days
- Examples of completed rapid syntheses.

Available at: [www.mcmasterhealthforum.org/policymakers/rapid-response-program](http://www.mcmasterhealthforum.org/policymakers/rapid-response-program)

**Strengths:**

- Reviews are supported by the Health Systems Evidence database ([www.healthsystemsevidence.org](http://www.healthsystemsevidence.org)), which include AMSTAR quality assessments.
- Providing a very clearly defined scope of what can be done in the available time.

**Challenges:**

- All 10-day and 30-day reviews undergo a ‘merit review’, which can slow the process down.
- Balancing the workload in times of high demand – having staff available to come in when needed.

**Future work:**

- To build a pan-Canadian Rapid Response network for policymakers and stakeholders that removes some, or all, of the user-pay requirement outside of Ontario.
- To include rapid syntheses conducted by the McMaster Health Forum and other groups as a new type of document in the Health Systems Evidence database.
- To conduct an evaluation of the service.

**Sources:**

Dr Mike Wilson, Assistant Director, McMaster Health Forum, McMaster University, Hamilton, Canada

Website: <https://www.mcmasterhealthforum.org/policymakers/rapid-response-program>

References: [2-4]

## Sax Institute Evidence Check program

The Sax Institute is a not-for-profit organization that is independent of any one university or research group.  An Evidence Check rapid review is a concise summary of evidence that answers specific policy questions presented in a policy-friendly format. The Evidence Check program began in 2006 and was a joint initiative with the New South Wales Ministry of Health. The service is available to any health-related organizations, though the majority of work has been with government departments and NGOs in Australia. Reviews are conducted on a user-pays basis and usually take 3-4 months to complete.

The Sax Institute knowledge brokers work with the commissioning agency to clarify the review questions, scope and format, from which the research questions and proposal is prepared and agreed by the agency. Then researchers with expertise in the topic are recruited through several methods including: seeking expressions of interest through the Sax Institute website; emailing the Sax Institute members; and identifying researchers with relevant expertise through the RADAR database of researchers. The agency selects their preferred research team from all researchers who submit expressions of interest. Two separate contracts are used for each review: one with the government agency and one with the researchers (or university). The Sax Institute Knowledge Exchange staff provide project oversight – which involves regular contact with the research team and liaison between the policy agency and researchers. Knowledge Exchange staff work with the research team throughout the process to ensure that the review meets the needs of the agency and review the draft and final reports before sending them to the commissioning agency.

Final reports are produced in the Sax Institute style but standard headings are not used. Completed reviews are published on the Sax Institute website if the agency provides consent – most (but not all) do. Regular quality assurance interviews are conducted with policy makers and researchers at six months after the final review has been submitted. Over 180 Evidence Check reviews have been commissioned to date, with over 60 Evidence Check reviews published <https://www.saxinstitute.org.au/category/publications/evidence-check-library/>.

**Documents available to guide the reviews are:**

- A commissioning tool, which is completed by the agency to start the process.
- Guidelines to support the knowledge broker and reviewers.
- A detailed project brief with review questions, scope and format (prepared by the knowledge broker).
- All processes and templates are contained within the Evidence Check standard operating procedures.

**Strengths:**

- Extensive network of researchers through the Sax Institute members and through the Sax Institute’s RADAR database. This ensures that reviews are prepared by reviewers with expertise in the topic who are able to interpret evidence and provide recommendations on applicability to the local setting if required: <https://www.saxinstitute.org.au/our-work/knowledge-exchange/radar/>.
- The knowledge brokering process is well received by the commissioning agency and produces clearer review questions.
- Skilled staff to manage the process

**Challenges:**

- There is always a difference in perspective of ‘rapid’ between policy agencies and reviewers.
- Time and expertise is required to manage this process. If it is a new research team more support is usually needed from Sax Institute staff.

**Future work:**

Developing a formal training program for researchers.

**Sources:**

Ms Sian Rudge, Head, Knowledge Exchange Division, Sax Institute, Sydney, NSW, Australia

Website: <https://www.saxinstitute.org.au/our-work/knowledge-exchange/evidence-check/>

## Regional East African Community Health (REACH) Policy Initiative, Uganda

This rapid response program is supported by the SURE project^[[1]](#footnote-1)^ and is based at Makerere University in Kampala, Uganda. It was set up in 2010 as a donor-funded project but will be absorbed into the Uganda National Health Research Organization at the end of the project period to ensure its sustainability. The first 28 months of operation of the service has been evaluated and the program was shown to be feasible and acceptable to policymakers in a low-income country [5]. The service is coordinated by hired research staff that keep in regular contact with policymakers and health systems stakeholders. They are supported by a wide network of researchers in and outside the region.

Staff receive questions from policy makers and take them through a process of question clarification to ensure that the question is clear and answerable within 28 days. Staff then search for evidence related to the query, appraise it, contextualize and summarize it. This summary is then reviewed by local and international experts on the given subject. Following this, staff prepare a short brief of usually four pages maximum, with clear key messages to submit to the policy maker. A response can also be prepared in a shorter timeframe (e.g. less than five days using a modified process). The scope of the service is limited to health systems questions and health technology assessments. The service was offered to policy and decision makers at ministries of health, districts or local governments, civil society organizations, health-related agencies, the private sector, and legislators. In practice, it has been most used by the Ministry of Health at the central headquarters (possibly due to its proximity to the service), agencies and NGOs; with no response from the districts. In the first 28 months of operation 60 questions were answered.

**Other documents available to guide the reviews are:**

### SURE guides for preparing evidence-based policy briefs: <http://www.who.int/evidence/sure/guides/>

- SURE template for rapid responses: <http://www.who.int/evidence/sure/rapidresponses/en/>
- Other guides, checklists and resources: see additional file from Mijumbi and colleagues [5].

**Strengths:**

This is one of few services that has published an evaluation of its activities. The service has been shown to increase the confidence of policy makers involved in the policy making process. Users have been satisfied with the service. Regular contact between the policy makers and the researchers at the service was an important factor in response to, and uptake of the service.

**Challenges:**

- Getting the policy makers to use the service at the start but users often later referred colleagues.
- The absence of reliable and fast internet connections, or access to databases and full text papers.
- Requires investment in human and other resources.
- Getting staff with the right mix of skills and qualifications to work on the service.

**Future work:**

Institutionalizing the service and ensuring its sustainability following the end of the project period.

**Source:** Mijumbi and colleagues [5]

# References

1. Khangura S, Konnyu K, Cushman R, Grimshaw J, Moher D. Evidence summaries: the evolution of a rapid review approach. Systematic Reviews. 2012;1:10.

2. Wilson MG, Lavis JN, Gauvin FP. Issue brief: Developing a ‘rapid-response’ program for health system decision-makers in Canada. 7 March 2014. Hamilton, Canada: McMaster Health Forum; 2014.

3. Wilson MG, Lavis JN, Gauvin FP. Dialogue summary: Developing a ‘rapid-response’ program for health system decision-makers in Canada. 7 March 2014. Hamilton, Canada: McMaster Health Forum, 2014.

4. Wilson MG, Lavis JN, Gauvin FP. Developing a rapid-response program for health system decision-makers in Canada: findings from an issue brief and stakeholder dialogue. Systematic Reviews. 2015;4:25.

5. Mijumbi RM, Oxman AD, Panisset U, Sewankambo NK. Feasibility of a rapid response mechanism to meet policymakers' urgent needs for research evidence about health systems in a low income country: a case study. Implementation Science. 2014;9:114.

1. Supporting the Use of Research Evidence (SURE) for policy in African health systems – a collaborative project that builds on and supports the Evidence-Informed Policy Network (EVIPNet) in Africa and the Regional East African Community Health (REACH) Policy Initiative. SURE is funded by the European Commission’s 7th Framework Programme. [www.evipnet.org/sure](http://www.evipnet.org/sure). [↑](#footnote-ref-1)
